# *Ex vivo* MRI and histopathology detect novel iron-rich cortical inflammation in frontotemporal lobar degeneration with tau versus TDP-43 pathology

**DOI:** 10.1016/j.nicl.2021.102913

**Published:** 2021-12-14

**Authors:** M. Dylan Tisdall, Daniel T. Ohm, Rebecca Lobrovich, Sandhitsu R. Das, Gabor Mizsei, Karthik Prabhakaran, Ranjit Ittyerah, Sydney Lim, Corey T. McMillan, David A. Wolk, James Gee, John Q. Trojanowski, Edward B. Lee, John A. Detre, Paul Yushkevich, Murray Grossman, David J. Irwin

**Affiliations:** a Radiology, Perelman School of Medicine, University of Pennsylvania, United States; b Neurology, Perelman School of Medicine, University of Pennsylvania, United States; c Pathology and Laboratory Medicine, Perelman School of Medicine, University of Pennsylvania, United States

**Keywords:** Frontotemporal lobar degeneration, Alzheimer’s disease, Iron, *Ex vivo* MRI, Histopathology

## Abstract

Frontotemporal lobar degeneration (FTLD) is a heterogeneous spectrum of age-associated neurodegenerative diseases that include two main pathologic categories of tau (FTLD-Tau) and TDP-43 (FTLD-TDP) proteinopathies. These distinct proteinopathies are often clinically indistinguishable during life, posing a major obstacle for diagnosis and emerging therapeutic trials tailored to disease-specific mechanisms. Moreover, MRI-derived measures have had limited success to date discriminating between FTLD-Tau or FTLD-TDP. T_2_*-weighted (T2*w) *ex vivo* MRI has previously been shown to be sensitive to non-heme iron in healthy intracortical lamination and myelin, and to pathological iron deposits in amyloid-beta plaques and activated microglia in Alzheimer’s disease neuropathologic change (ADNC). However, an integrated, *ex vivo* MRI and histopathology approach is understudied in FTLD. We apply joint, whole-hemisphere *ex vivo* MRI at 7 T and histopathology to the study autopsy-confirmed FTLD-Tau (n = 4) and FTLD-TDP (n = 3), relative to ADNC disease-control brains with antemortem clinical symptoms of frontotemporal dementia (n = 2), and an age-matched healthy control. We detect distinct laminar patterns of novel iron-laden glial pathology in both FTLD-Tau and FTLD-TDP brains. We find iron-positive ameboid and hypertrophic microglia and astrocytes largely in deeper GM and adjacent WM in FTLD-Tau. In contrast, FTLD-TDP presents prominent superficial cortical layer iron reactivity in astrocytic processes enveloping small blood vessels with limited involvement of adjacent WM, as well as more diffuse distribution of punctate iron-rich dystrophic microglial processes across all GM lamina. This integrated MRI/histopathology approach reveals *ex vivo* MRI features that are consistent with these pathological observations distinguishing FTLD-Tau and FTLD-TDP subtypes, including prominent irregular hypointense signal in deeper cortex in FTLD-Tau whereas FTLD-TDP showed upper cortical layer hypointense bands and diffuse cortical speckling. Moreover, differences in adjacent WM degeneration and iron-rich gliosis on histology between FTLD-Tau and FTLD-TDP were also readily apparent on MRI as hyperintense signal and irregular areas of hypointensity, respectively that were more prominent in FTLD-Tau compared to FTLD-TDP. These unique histopathological and radiographic features were distinct from healthy control and ADNC brains, suggesting that iron-sensitive T2*w MRI, adapted to *in vivo* application at sufficient resolution, may eventually offer an opportunity to improve antemortem diagnosis of FTLD proteinopathies using tissue-validated methods.

## Highlights

• Comparative study of whole-hemisphere *ex vivo* T2*-weighted MRI and histopathology.
• Sample of FTLD-Tau and FTLD-TDP subtypes with reference to healthy and AD brain.
• Novel focal upper cortical-layer iron-rich pathology distinguishes FTLD-TDP from clinically-similar FTLD-Tau and AD.
• Distinct novel iron-rich FTLD-Tau pathology in mid-to-deep cortical-layers and WM.
• T2*-weighted MRI signatures offer *in vivo* biomarker targets for FTLD proteinopathy.

## Graphical abstract

**Figure:**
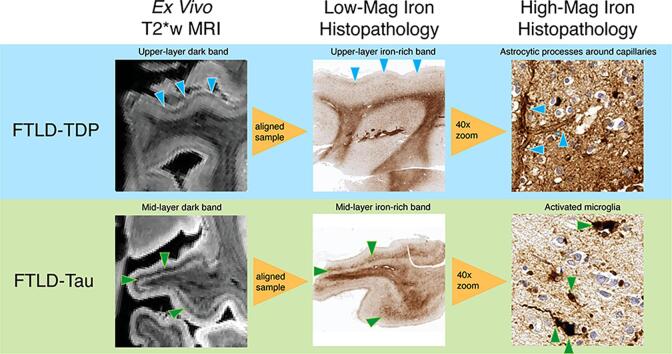

## 1 Introduction

Frontotemporal lobar degeneration (FTLD) is an understudied family of age-associated neurodegenerative proteinopathies that encompasses a variety of progressive frontotemporal dementia (FTD) clinical syndromes and is the most common cause of young-onset dementia. (Rossor et al., 2010) Roughly 95% of FTLD is caused by one of two distinct proteinopathies: tauopathies (i.e., FTLD-Tau) or TDP-43 proteinopathies (i.e., FTLD-TDP). (Mackenzie et al., 2010) Despite the distinct postmortem neuropathology that distinguishes these proteinopathies microscopically, it is not currently possible to accurately detect and differentiate these pathologies during life. (Irwin et al., 2015) Indeed, while some frontotemporal dementia (FTD) clinical syndromes have group-level statistical associations with one of these proteinopathies, the majority of FTD syndromes do not reliably predict pathology. (Irwin et al., 2015, Gorno-Tempini et al., 2011, Höglinger et al., 2017, Giannini et al., 2019, Irwin et al., 2018, Perry et al., 2017, Spinelli et al., 2017) Moreover, while various degenerative processes can be detected using structural, (McKiernan and O’Brien, 2017, McMillan et al., 2014, Rohrer et al., 2009, Whitwell et al., 2005, Whitwell et al., 2015, Perry et al., 2017), diffusion, (McMillan et al., 2014, Whitwell et al., 2010, Illán-Gala et al., 2019), and spectroscopic MRI, (Maul et al., 2020, Murley et al., 20202020), as well as PET, (Kim et al., 2019, Whitwell et al., 2020); there is currently no method to directly sensitize traditional MRI, or any other *in vivo* imaging technology, including molecular imaging, to the specific protein inclusions or pathological features that would allow *in vivo* discrimination of patients with tau and/or TDP-43 inclusions in FTLD. In this report, we explore the novel combination of histopathology and T_2_*-weighted (T2*w) *ex vivo* 7 T MRI for the purpose of developing a more reliable, data-driven approach to diagnosis of pathology in FTLD spectrum disorders.

MRI-based studies of FTLD have largely focused on localizing neurodegeneration through quantification of cortical grey matter thinning and/or coherence of white matter fibers, (Gordon et al., 2016, Meeter et al., 2017). Both methods reveal group-level patterns of atrophy and disruption of neurocognitive networks that map to clinical symptoms. (Rohrer et al., 2009, Collins et al., 2017, Grossman et al., 2013, Seeley et al., 2009) However, while gross regional patterns of neurodegeneration are associated at a group level with specific FTLD pathologies in autopsy data, (Giannini et al., 2019, Rohrer et al., 2011, Spinelli et al., 2017, Whitwell et al., 2011, Perry et al., 2017), patient-level prediction of pathology using these methods remains elusive. (Whitwell and Josephs, 2012) Thus, *in vivo*, individual-patient level prediction of pathology is a major impediment and unmet need for development of disease-modifying therapies. (Irwin et al., 2015, Meeter et al., 2017) In contrast, pathology-based studies of FTLD have suggested a variety of disparate microscopic features across tau-(Dickson et al., 2011, Irwin et al., 2016, Kovacs et al., 2020) or TDP-43-mediated neurodegeneration, (Mackenzie and Neumann, 2020, Sakae et al., 2019). However, microscopic studies are necessarily restricted in spatial scope due to the nature of histopathologic sampling and analysis.

*Ex vivo* MRI provides ultra-high-resolution imaging of fine structures over large sections of tissue. Previous work in other disorders has demonstrated significant correlations between T2*w *ex vivo* MRI at 7 T and histopathology, particularly in the mapping of myelin and iron deposits in cortical laminae. (Bulk et al., 2020, Fracasso et al., 2016, Fukunaga et al., 2010, Hametner et al., 2018, Wallace et al., 2016) Indeed, these two sources of contrast often overlap, as oligodendrocytes are a major source of non-heme iron in the brain due to the large metabolic demands for myelination. (Cheli et al., 2020) Joint MRI/pathology studies have particularly focused on Alzheimer’s disease neuropathologic change (ADNC) (Bulk et al., 2020, Benveniste et al., 1999, Bulk et al., 2018, Bulk et al., 2018, Gong et al., 2019, Meadowcroft et al., 2009, van Rooden et al., 2009, Zeineh et al., 2015) and amyotrophic lateral sclerosis (ALS) (Kwan et al., 2012, Meadowcroft et al., 2015, Pallebage-Gamarallage et al., 2018, Wang et al., 2020), both of which have been described producing specific and localized distributions of pathological intracortical iron. However, in FTLD, joint MRI/pathology has been largely limited to focused study of the basal ganglia. (De Reuck et al., 2014, Foroutan et al., 2013, Massey et al., 2017)

In this work we explore the combined use of histopathology and T2*w *ex vivo* 7 T MRI of whole brain hemispheres to 1) evaluate the sensitivity of T2*w MRI to detect and distinguish microscopic pathologic features of FTLD within cortical laminae and subjacent WM, and 2) demonstrate utility of MRI-guided histopathology to locate and typify focal pathologic features in FTLD. Previous histopathological studies suggest relative bilaminar distribution of tau inclusions, (Armstrong et al., 1999, Armstrong and Cairns, 2012, Irwin et al., 2016) and gliosis (Kersaitis et al., 2004, Schofield, 2003, Hasegawa et al., 2018, Cooper et al., 1996) with greater WM degeneration60, (Giannini et al., 2021) in FTLD-Tau. In contrast, FTLD-TDP demonstrates greater relative upper cortical-layer TDP-43 inclusions, (Armstrong et al., 2012, Armstrong et al., 2013, Armstrong and Cairns, 2012), degeneration and gliosis, (Lant et al., 2014, Sakae et al., 2019, Taipa et al., 2017). Therefore, we hypothesized T2w* MRI would reveal distinct laminar features of greater relative deep cortical layer and adjacent WM disease in FTLD-Tau compared to greater relative upper layer pathology in FTLD-TDP. Our findings’ laminar distribution were in concordance with this hypothesis, and, moreover, we describe below novel iron-rich patterns of gliosis in FTLD that highlight potential distinct mechanisms of neuroinflammation between proteinopathies.

## 2 Materials and methods

### 2.1 Patients and neuropathological diagnosis

Patients selected for study were evaluated at the Penn Frontotemporal Degeneration Center (FTDC). Clinical diagnosis was performed in a weekly, multidisciplinary consensus panel using published clinical research criteria for FTD. (Gorno-Tempini et al., 2011, Höglinger et al., 2017, Rascovsky et al., 2011) Autopsy was performed at the Penn Center for Neurodegenerative Disease Research (CNDR). Neuropathological diagnosis was performed using tissue from the non-scanned hemisphere by experienced neuropathologists (EBL, JQT) using well characterized antibodies (Toledo et al., 2014) and current diagnostic criteria. (Mackenzie et al., 2011, Mackenzie et al., 2010, Montine et al., 2012) We included only sporadic FTLD patients, genotyped based on pedigree-analysis, using a custom targeted next-generation sequencing panel for neurodegenerative diseases including *MAPT, GRN* and repeat-primed PCR for *C9orf72*, as previously described. (Irwin et al., 2018)

In our initial discovery cohort, we examined 3 FTLD-Tau brains, and 2 FTLD-TDP brains, 1 Alzheimer’s disease neuropathologic change (ADNC) brain, and 1 age-matched healthy control brain to capture the heterogeneity of FTLD. After analysis of our discovery cohort, 3 additional samples (1 FTLD-Tau, 1 FTLD-TDP and 1 ADNC) were added as a replication cohort, to confirm novel MRI-detected histopathological features in these pathologic groups.

Tauopathies can be subdivided into the prominent isoform of tau present in inclusions (i.e. 3- and 4-repeat, [3R, 4R]), (Mackenzie et al., 2010) and our sample contained both 3R and 4R pathologies. Similarly, FTLD-TDP can be subdivided into subtypes TDP-A, -B, and -C, (Mackenzie et al., 2011) and our sample contained both TDP-A and -C. Patient demographics and clinical characteristics are summarized in Table 1.

**Table 1:** Summary of demographic data with histopathologic and neuropsychological testing results for both discovery and replication cohorts.

		Discovery Cohort							Replication Cohort		
	Patient #	1	2	3	4	5	6	7	8	9	10
Demographics	Syndrome	none	bvFTD	svPPA/bvFTD	bvFTD	naPPA/CBS	PSPS/naPPA	bvFTD	bvFTD	bvFTD/svPPA	naPPA/CBS
	Neuropath	PART/CVD	AD-High	TDPC	TDPA	GGT	PSP	PiD	AD-High^†^	TDPC	GGT
	ABC	A0B1C0	A2B3C3	A1B0C0	A0B2C0	A2B0C0	A2B0C3	A1B0C0	A3B3C3	A1B0C0	A1B2C0
	Sex	F	M	M	F	M	M	M	F	M	F
	Age at death	75	75	70	73	76	74	74	66	65	74
	Disease duration (y)		6	7	14	4	7	6	10	5	6
	Age-first visit		72	64	62	73	68	71	61	61	69
	Age-last visit		74	70	70	75	72	73	63	64	72
	# visits		5	12	10	6	8	5	5	7	9
	Onset- first visit interval (y)		3	1	3	2	2	2	5	1	2
	Last visit-autopsy interval (y)		2	<1	4	1	1	2	3	1	2
	Hemi. sampled	R^#^	R	L	R	L	L	R^#^	R	R	L
	Brain weight (g)	1188	1258	1036	995	1316	1331	1217	911	1208	1179
	Post-mortem interval (hours)	12	14	17	21	18	11	24	4	13	19
	Fix. time (days)	87	167	114	59	165	93	626	57	132	39
Neuropsych. Testing	MMSE-first		22	27	29	29	26	26	0	19	28
	MMSE-last		4	7	16	2	14	16	0	8	20
	CDR SOB-first		9.5	1.5	12.5*	7	4	13	22*	5.5	2
	CDR SOB-last		10	21	17*	12.5	11	13.5	24*	19	4
	VF		6	5	10	5	5	8	NA	13	4
	Animals		7	9	10	11	6	16	NA	0	10
	BNT		8	20	18	27	28	24	NA	1	28
	Craft Memory		0	9	NA	12	9	13	NA	4	6
	Benson copy		10	17	NA	14	6	17	NA	17	17
	Benson Recall		2	0	NA	8	6	3	NA	4	12
Behavior	Disinhibition		Onset	Onset	Onset	–	6	Onset	5	Onset	4
	Apathy		Onset	Onset	4	5	–	Onset	Onset	Onset	4
	Loss of empathy		Onset	Onset	Onset	–	6	Onset	Onset	Onset	–
	Perseverative		Onset	Onset	Onset	–	–	Onset	Onset	Onset	–
	Hyperoral		Onset	Onset	Onset	5	–	Onset	Onset	Onset	–
Lang	Non-fluent		5	–	–	Onset	4	–	5	–	Onset
	Gram. Errors		5	–	–	Onset	4	Onset	5	–	Onset
	Semantic		4	Onset	–	–	–	–	5	Onset	–
	Repetition		Onset	–	–	5	–	–	5	Onset	4
Mot	Parkinsonism		5	6	8	Onset	Onset	Onset	7	–	4
	Gaze Paralysis		–	–	–	–	Onset	–	–	–	–
	Apraxia		–	–	–	Onset	Onset	–	5	–	Onset

Syndrome: bvFTD = behavioral variant of FTD, svPPA = semantic variant of primary progressive aphasia, naPPA = nonfluent agrammatic variant of primary progressive aphasia, CBS = corticobasal syndrome, PSPS = progressive supranuclear palsy syndrome

Neuropath: PART = primary age-related tauopathy, CVD = cerebrovascular disease (left hemisphere remote infarct and arteriovenous malformation), AD-High = High level of Alzheimer’s disease neuropathological change, TDPA = TDP-43 proteinopathy type A, TDPC = TDP-43 proteinopathy type C, GGT = globular glial tauopathy, PSP = progressive supranuclear palsy tauopathy, PiD = Pick’s disease tauopathy, ^†^ = mild Lewy body co-pathology restricted to amygdala (i.e. amygdala-only stage Lewy body disease)

ABC: Amyloid Thal stage/ Braak AD Tau Stage/ CERAD plaque stage

# visits: Number of patient visits at Penn FTDC with structured assessment of clinical features of FTD

Onset-first visit interval: Years between first visit and reported onset of disease

Last visit-autopsy interval: Years between most recent visit and autopsy in years

Hemi. sampled: Hemisphere sampled for this study. ^#^ = only frontal lobe available for MRI.

Fix. time: Fixation time

Neuropscych. Testing section: Tests as described below, with exception of entries marked NA due to level of impairment at time of testing.

MMSE-first: First available Folstein Mini Mental status examination® score (max = 30).

MMSE-last: Most recent Folstein Mini Mental status examination® score (max = 30)

CDR SOB-first: First available extended clinical dementia rating scale (CDR®+NACC-FTLD) sum of boxes score (max = 24). * = CDR scoring performed retrospectively from chart review.

CDR SOB-last: Most recent extended clinical dementia rating scale (CDR®+NACC-FTLD) sum of boxes score (max = 24). * = CDR scoring performed retrospectively from chart review.

VF: Baseline visit score of verbal fluency; total number of words beginning with letter “F” in 60 s

Animals: Baseline visit score of category fluency; total # of words in 60 s in category of animals

BNT: Baseline visit score on 30 item Boston Naming Test

Craft Memory: Baseline visit score on craft story verbal memory test; total story units recalled with verbatim scoring (max = 44)

Benson copy: Baseline visit total score for copy of Benson figure (total = 17)

Benson recall: Baseline visit total score for visual memory delayed (total = 17)

Behavior, Language, and Motor sections: Clinical features from the medical record were recorded as the first report for onset of sign (Onset = presenting feature of disease within first three years; numbers indicate years after reported onset where noted, - = not present in the record). Behavior features (Disinhibition, Apathy, Loss of empathy, Perseverative behavior, and Hyperorality) defined in current clinical diagnostic criteria for bvFTD. (Rascovsky et al., 2011) Language features (Non-fluent speech, Grammatical errors, Semantic, Repetition) defined in current diagnostic criteria for PPA. (Gorno-Tempini et al., 2011) Motor features included Parkinsonism = presence of signs of bradykinesia, rigidity, postural instability or tremor; Gaze paralysis = occulomotility disorder for vertical saccades; and limb Apraxia.

Clinical data for features of FTD syndromes were extracted from the medical record by an experienced investigator (DJI) and recorded as the time of onset reported for each symptom or sign, any clinical feature reported within the first 3 years of disease was considered part of the presenting syndrome, as we have done previously. (Irwin et al., 2016, Giannini et al., 2021) Neuropsychological testing data was obtained from Penn Integrated Neurodegenerative disease database. (Xie et al., 2011) All procedures were performed in accordance with Helsinki criteria and an informed consent procedure obtained in accordance with the University of Pennsylvania Institutional Review Board.

At the time of autopsy, one hemisphere was selected for standard neuropathological sampling from fresh tissue for diagnostics and frozen storage of remaining tissue as previously described, (Toledo et al., 2014) while the other hemisphere was immersed in 10% neutral buffered formalin for at least 30 days prior to imaging as below (Min = 59, Max = 626, Mean = 187 days).

To harmonize with ongoing projects at our center, some hemisphere samples used for scanning were from the frontal lobe only and/or had 1.5 cm fresh samples taken prior to fixation for bilateral assessments of pathology in FTD, as previously described. (Giannini et al., 2019)

Finally, for further histopathological replication, we selected 19 patients with FTLD-Tau and 11 patients with FTLD-TDP, all without significant ADNC co-pathology and with available tissue from our brain bank. These legacy samples did not have *ex vivo* MRI, but were used to test generalizability of our histopathological findings of unique laminar patterns of novel iron-rich gliosis in forms of FTLD in select regions identified in our *ex vivo* data.

### 2.2 *Ex vivo* 7 T MRI

Samples were placed in Fomblin (California Vacuum Technology; Freemont, CA), a proton-free fluid with volume magnetic susceptibility close to that of tissue. Samples were enclosed in either custom-build cylindrical holders or plastic bags, and then left to rest for at least two days to allow air bubbles to escape from the tissue. Depending on their size, samples were scanned using either a custom-built small solenoid coil or a custom-modified quadrature birdcage (Varian, Palo Alto, CA, USA) coil. These transmit/receive coils were attached to a two-channel transmit-receive adapter (Stark Contrast, Erlangen, Germany). The smallest coil was chosen that could hold each sample. In particular, while whole intact hemispheres all were scanned with the larger birdcage coil, one of our samples (Patient #7) consisted of a partial hemisphere and so was fit in a smaller sample holder with less fomblin to prevent the sample from moving during scanning. To ensure optimal coil matching, we used our smaller custom coil, optimized for use with the small sample-holder, for this partial-hemisphere sample. Once loaded into a coil, the sample was placed into our whole-body 7 T scanner (MAGNETOM Terra, Siemens Healthineers, Erlangen, Germany) with plastic shims used under the coils to raise them off the table and position the sample near isocenter.

MRI data were acquired with a 3D-encoded, 8-echo gradient-recalled echo (GRE) sequence with non-selective RF pulses. To maintain readout polarity and minimize distortions due to field inhomogeneity, each readout was followed by a flyback rephrasing gradient. The final echo was followed by an additional completely rephrased readout to measure frequency drifts. Each line of k-space was acquired with multiple averages sequentially before advancing to the next phase-encode step. Common parameters for the sequence were: 280 μm isotropic resolution, 25° flip angle, 60 ms repetition time (TR), minimum echo time (TE) 3.48 ms, echo spacing 6.62 ms, bandwidth 400 Hz/px. The field of view was adapted to each sample, and subsequently TRs and TEs were slightly modified based on the necessary readout duration. Total scan times were 8–10 h for each sample. Matrix size and number of averages acquired for each sample are shown in Supplementary Table 5.

Images were reconstructed using the vendor’s on-scanner reconstruction software which automatically corrected the global frequency drift, combined the signal averages in k-space, and produced magnitude images for each echo. After its MRI session, each sample was rinsed in formalin and then stored at room temperature in sealed bags for histopathological processing.

After inspecting the 8 individual echo images, we determined that an echo time of roughly 20 ms provided strong T2*-weighted cortical laminar contrast while maintaining excellent SNR and minimizing local susceptibility induced drop-outs (*e.g.*, due to air bubbles). We performed all subsequent analyses only on these 20 ms single-echo images, leaving aside the remaining 7 echos from our present analyses. This choice of TE is consistent with some previous work using magnitude images directly, (Fracasso et al., 2016, Kenkhuis et al., 2019) while slightly shorter than that chosen by others. (Bulk et al., 2020, Bulk et al., 2018, Bulk et al., 2018, Kwan et al., 2012) SNR measures for each pathology-sampled region (sampling process described below), are presented in Supplementary Table 5.

At each pathology-sampled region (sampling process described below), MR images were rated by an experienced investigator (MDT). We quantified the relative contrast between pairs of neighboring structures using a range (-2, +2), where + 2 indicates the first structure in the pair is substantially brighter than the second, −2 indicates the first structure in the pair is substantially darker than the second, and 0 indicates no apparent contrast between the structures (see Supplementary Tables 3 and 4 for complete rankings). In addition, to provide ratings similar to those in our histopathological analysis (see below) we rated the degree of focal abnormal hypointensity on an ordinal scale from 0 (no abnormality) to 3 (frank abnormality), without attempting to account for differences in texture, etc. that are discussed in the remainder of these results. Within each region, these “MRI abnormality” ratings were performed for upper grey matter, deep grey matter, and subjacent white matter (see Supplementary Table 1 for complete rankings).

### 2.3 Histopathologic sampling

All discovery cohort patient samples were first systematically sampled in the Penn Digital Neuropathology Lab using an atlas-based approach in key neocortical regions implicated in FTD, (Giannini et al., 2019, Irwin et al., 2018) including orbitofrontal cortex (OFC, Brodmann area (BA) 11), anterior temporal cortex (ATC, BA 20 with the exception of patients #3, #8, and #9, who only had tissue available for histology from adjacent BA 38 obtained fresh at autopsy), inferior prefrontal cortex (IPFC, BA 45) and primary motor (BA 4) known to be an area of high pathology in PSP. (Kovacs et al., 2020) We additionally sampled primary somatosensory (BA 3) as a relative negative control region that is generally less-affected in FTLD.

Critically, in reviewing our MRI data, we noted unique focal cortical features outside of our standard sampling in some of our discovery cohort patients. To further investigate pathologic-imaging correlations, we additionally sampled these specific regions (specific MRI-guided regions are discussed for each sample in Results).

During both the atlas- and MRI-guided sampling, FreeView 6.0 (Athinoula A. Martinos Center for Biomedical Imaging, https://surfer.nmr.mgh.harvard.edu/fswiki/FreeviewGuide) was used to view the MR images and generate 3D surface models of the hemispheres on a bench-side computer. By matching gyral patterns on the hemisphere sample to the 3D surface model and MR images, the sampling team (DO, DJI, MDT) achieved consensus on the correspondence between imaging coordinates and histology sample locations. Samples, roughly 1.5 cm × 1.5 cm × 0.5 cm, were taken with cuts normal to the pial surface.

All samples were processed and embedded in paraffin, then sectioned into 6 um sections in the Penn Digital neuropathology lab for histological evaluation. (Irwin et al., 2016) Adjacent sections from each tissue sample were stained for healthy myelin using luxol-fast blue with hematoxylin and eosin to visualize GM (LFB), (McMillan et al., 2013) Meguro method for Perl’s Fe (iron) stain with DAB amplification, (Meguro et al., 2007, van Duijn et al., 2013) amyloid-beta (Nab228, CNDR), (Lee et al., 2003) ferritin light chain (Abcam; Catalogue No ab69090), microglia (IBA-1; Santa Cruz Biotechnology Inc, Dallas, TX; Catalogue No sc-32725) and activated astrocytes (GFAP; Dako; Santa Clara, CA; Catalogue No Z0334). Moreover, sections for FTLD-Tau and ADNC were also immunostained for phosphorylated tau (AT8; Fisher, Waltham, MA; Catalogue No. ENMN1020) (Mercken et al., 1992) and FTLD-TDP for phosphorylated TDP-43 (p409.410; Protein Tech, Rosemont, IL; Catalogue No 66318–1-Ig) (Neumann et al., 2009) to identify protein inclusions characteristic of these disorders. All sections were counterstained with hematoxylin. To ensure specificity and reproducibility of chemical staining, sections were re-stained in three separate staining batches for LFB and iron stain.

Slides were reviewed by an experienced investigator (DJI) and rated for key histopathological features on a standardized 0–3 ordinal scale (i.e. none, rare, mild, moderate, severe) (Montine et al., 2012) including: 1) density of tau/TDP-43, glial and iron-rich pathology in supragranular GM upper (layers I-III), infragranular deep GM (infragranular IV-VI), juxtacortical WM enriched with U-fibers and adjacent relatively deep WM; 2) vacuolization and neuronal loss in upper and deep GM layers; and 3) the graded presence of intracortical WM (i.e., bands of Baillarger) and adjacent juxtacortical and relatively deeper WM in LFB and Fe-stained sections.

In our replication cohort with *ex vivo* MRI, samples were selected based on the region of greatest pathology in the matched discovery cohort cases, drawn from among the standard samples acquired at autopsy prior to scanning (anterior temporal cortex in BA 38 and primary motor cortex) and matched to adjacent tissue on MRI.

In our histopathological replication cohort of legacy autopsy samples, we used Meguro method to iron stain similarly standardized fresh-sampled regions to confirm our observations of unique iron-rich gliosis from ex vivo MRI in primary motor cortex for PSP tauopathy patients, mid-frontal cortex for Pick’s disease tauopathy, anterior temporal lobe (BA38) for FTLD-TDP type C patients and orbitofrontal cortex for FTLD-TDP type A. Slides were rated in upper and deep GM for the presence of characteristic iron rich gliosis morphologies identified in *ex vivo* cohort (i.e. astrocytic processes enveloping small vessels and hypertrophic microglia) on the same 0–3 ordinal scale.

### 2.4 Data and code availability

All MRI imaging data that support this study are openly available via Dryad at https://doi.org/10.5061/dryad.4tmpg4f8r. All histopathology data that supports this study are contained in the supplementary materials and tissue is available upon reasonable request from the authors, conditional on establishing a formal data sharing agreement with the University of Pennsylvania. No locally developed software was used in this study.

## 3 Results

### 3.1 Semiquantitative results of MRI and histopathology

**Fig. 1:**
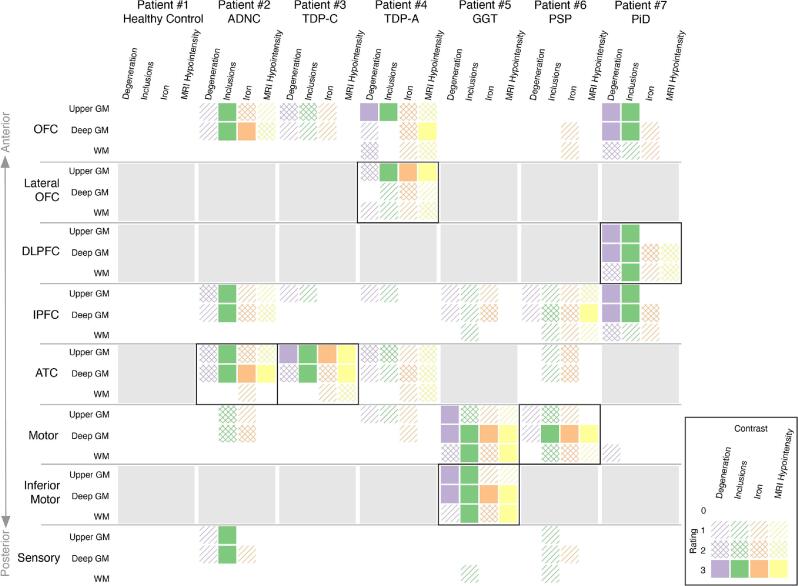
Semiquantitative summary of discovery cohort histopathology and MRI ratings. Ordinal ratings of histopathology and MRI hypointensity are presented in a tabular display (legend at bottom right). Columns represent individual hemisphere samples, while rows represent individual anatomical regions, roughly organized anterior-to-posterior. Within each column, four sub-columns represent rankings of (*purple*) neurodegeneration (neuron loss in GM, myelin loss in WM), (*green*) pathological inclusions (Tau immunoreactivity for FTLD-Tau and AD; TDP-43 immunoreactivity for FTLD-TDP patient samples) , (*orange*) pathological iron, and (*yellow*) T2*w hypointense abnormalities. Within each row, three sub-rows represent sampling depth: upper GM, deep GM, and subjacent WM. Within each cell, the ordinal rating is designed by the degree of filing: (*blank*) 0 = none/normal, (*single hatch*) 1 = mild, (*cross-hatch*) 2 = moderate, (*filled*) 3 = severe. Comparing along sub-columns within a cell shows correlation between histopathology and MRI. Comparing along sub-rows within a cell shows disease spread across the depth of the tissue. Comparing cells within a column shows spread of disease across regions of the brain. Regions displayed in subsequent figures are surrounded by black boxes. Areas not sampled for study are shaded in grey. (For interpretation of the references to colour in this figure legend, the reader is referred to the web version of this article.)

Fig. 1 depicts the main histopathological and MRI findings in our discovery cohort. Overall, ordinal rankings of various indices of neurodegeneration correlated with each other and with iron-rich gliosis, both regionally (*e.g.*, orbitofrontal vs motor cortex) and within the cortical layers (*e.g.*, upper vs deep). The ordinal MRI hypointensity rankings show a similar spatial distribution to the pathology but were most consistent with pathological iron on histology.

Our results for pathological iron deposits on histology and T2*w MRI largely match expected spatial distributions of degeneration across the cortex, with regions of pathology broadly correlated with clinical/pathological variants (*e.g.*, predominantly anterior temporal degeneration in svPPA with FTLD-TDP C (Giannini et al., 2019) and ventral frontal degeneration in bvFTD with FTLD-TDP A (Giannini et al., 2021), compared to primary motor cortex in PSP (Kovacs et al., 2020) and GGT tauopathy(Ahmed et al., 2013)). The PiD tauopathy sample had less prominent iron-rich gliosis largely restricted to a focal area of dorsolateral prefrontal cortex, an area of high pathology in PiD (Irwin et al., 2016), along with widespread loss of intracortical myelin throughout the frontal neocortex. As MRI hypointensities in our data are generally linked to iron, our MRI scores more closely follow the iron ratings, and rather than the neurodegeneration or inclusion scores. This is discussed in more detail in sample-specific subsections below.

For brevity in the discussion of individual samples below, we will focus on the specific regions for each subject that had distinct MRI/histopathological findings compared to the healthy control and/or other patient regions. Please see Supplementary Tables 1–4 for full histopathological and MRI ratings. For each discovery subject below, we present a detailed visualization of a single region of focal pathology. For ease of comparison, these regions, along with matched regions in the replication cohort, are also displayed together as a single figure (see Fig. 8).

### 3.2 Patient #1 — Healthy control

As expected, T2*w MRI showed dark WM with lighter cortical GM that, in turn, was generally darkest in lower layers, with one or two distinct tangential bands generally observed, depending on the cortical region (Supplementary Fig. 1). Juxtacortical WM enriched with U-fibers generally showed a slight gradient of darker signal compared to deeper adjacent WM. Comparing with histology, these contrast differences were, as expected, produced by iron-containing myelinated fibers — radial fibers producing lower-layer cortical hypointensity and bands of Baillarger producing tangential hypointense “stripes”. There was also slightly greater density of iron-rich myelin in juxtacortical WM enriched for U-fibers around sulcal depths compared to relatively deeper WM.

Consistent with the pathological staging data (Table 1) there was an absence of neurodegenerative disease-associated protein deposits in regions sampled. Microglia detected by IBA-1 were found throughout GM and WM showing a pattern of quiescent resting-type “ramified” morphology (Fig. 9, solid black arrowheads) and only rare iron- or ferritin-reactive activated microglia with a rod- or ameba-shaped morphology (Fig. 9, solid yellow arrowheads). (Bachstetter et al., 2015, Ito et al., 1998) Astrocytes detected by GFAP immunostain found mild sub-pial reactivity (Fig. 9, open black arrowheads) that seldom reached deeper than layer I and uniform astrocyte reactivity in WM, both largely in a “fibrous” morphology, as previously described in healthy brain. These morphologies contrasted with a more hypertrophic activated morphology and greater overall density of GM protoplasmic astrocytes detected by GFAP stain in neurodegeneration (Fig. 9, solid blue arrowheads). (Beach et al., 1989, Lundgaard et al., 2014)

### 3.3 Alzheimer’s disease neuropathologic Change, Behavioral-Variant Frontotemporal dementia syndrome

#### 3.3.1 Patient #2 (Discovery Cohort)

**Fig. 2:**
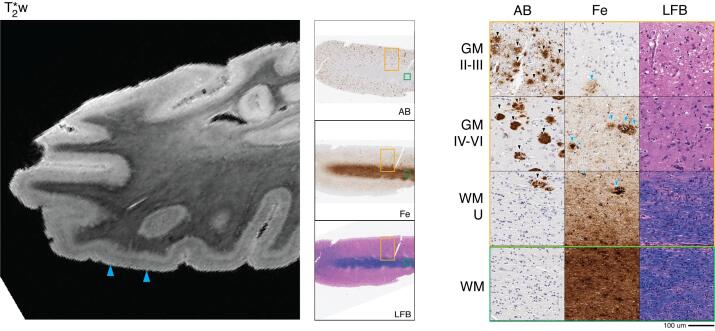
Alzheimer’s disease (patient #2) anterior temporal cortex MRI and pathology. *Left:* T2*w MRI of anterior temporal lobe (including sampled BA 20). *Center:* low-magnification (1x) view of tissue sample stained for amyloid-beta (AB), iron (Fe), and myelin (LFB). *Right:* high-magnification (20x) tissue sample stained for AB, Fe, and LFB (scale bar = 100 µm). Rows depict view in upper cortical layers (GM II-III), deep cortical layers (GM IV-VI), directly adjacent white matter enriched for cortical U-fibers (WM-U) and relatively deeper white matter (WM) from boxes outlined in low magnification view (orange box = cortical layers, green box = deep WM). There is widespread amyloid-beta plaque pathology (black arrowheads, AB) across cortical layers and in juxtacortical white matter along with severe neuronal loss across layers. T2*w MRI shows a widespread hypointense speckling pattern (blue arrowheads, T2*w) in mid-cortical layers which correlated iron deposits consistent in size and shape with neuritic senile plaques (blue arrowheads, Fe) and associated microglia (blue asterisks, Fe). (For interpretation of the references to colour in this figure legend, the reader is referred to the web version of this article.)

Consistent with previous reports in amnestic AD syndrome with ADNC pathology, on MRI we noted a pattern of large hypointense speckling in GM in middle to lower cortical layers. (Meadowcroft et al., 2009, Bulk et al., 2018, Bulk et al., 2018) This pattern was present throughout the neocortex and limbic cortex with relatively preserved patterns of GM lamination and WM architecture as found in the healthy control sample. In our pathology-sampled regions, this speckling pattern was most prominent in the ATC (Fig. 2, solid blue arrowheads).

Histopathologic examination of sampled tissue was consistent with neuropathological staging of high-level ADNC (Table 1) and revealed a high density of diffuse and neuritic amyloid-beta plaques and tau-positive tangles/threads largely confined to cortical GM.

Iron stain formed deposits that resembled a subset of iron-reactive amyloid-beta plaques and associated microglia in activated morphology (Fig. 2, solid blue arrowheads) that were most pronounced in anterior temporal and orbitofrontal cortex. The distribution and intensity of myelin and iron stain also indicated relative preservation of intracortical myelin and adjacent WM throughout most regions sampled, although there was reduced density in ATC.

#### 3.3.2 Patient #8 (Replication Cohort)

This replication patient also had high-level ADNC with antemortem bvFTD (Table 1). Based on our findings in patient #2, we focused our analysis on the ATC of patient #8. MRI findings were similar to patient #2, with large hypointense speckling in GM in middle to lower cortical layers throughout most of the neocortex, most prominent in ATC. Focused histopathological validation of the ATC found similar findings of mid-layer iron-rich plaques and associated iron-reactive microglia.

### 3.4 FTLD-TDP type C Neuropathology, Semantic variant PPA and Behavioral-Variant Frontotemporal dementia syndromes

#### 3.4.1 Patient #3 (Discovery Cohort)

**Fig. 3:**
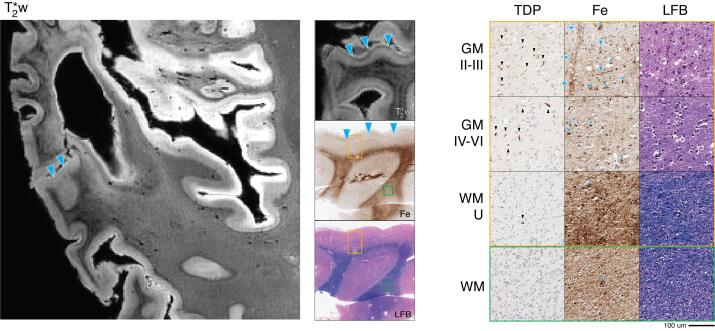
FTLD-TDP type C (patient #3) anterior temporal cortex MRI and pathology. *Left:* T2*w MRI of anterior temporal lobe (including sampled BA 38). *Center:* low-magnification (1x) view of tissue sample stained for iron (Fe) and myelin (LFB), with corresponding T2*w MRI slice. *Right:* high-magnification (20x) tissue sample stained for TDP-43 pathology (TDP), Fe, and LFB (scale bar = 100 µm). Rows depict view in upper cortical layers (GM II-III), deep cortical layers (GM IV-VI), directly adjacent white matter enriched for cortical U-fibers (WM-U) and relatively deeper white matter (WM) from boxes outlined in low magnification view (orange box = cortical layers, green box = deep WM). There is prominent TDP-43 pathology (black arrow heads, TDP) across cortical layers and most abundant in upper layers along with severe neuronal loss and vacuolization (LFB). T2*w MRI shows a hypointense upper-layer band (blue arrowheads, T2*w) and diffuse speckling which correlated with iron deposits resembling astrocyte processes surrounding capillaries (blue arrowheads, Fe) and activated microglia (blue stars, Fe). Outside of the upper-layer band, there is an absence of intracortical contrast on MRI (T2*w), and rare myelin fibers on histology (LFB). Adjacent white matter signal is heterogenous on MRI (T2*w) corresponding to mild reduction in relative deep white matter myelin (LFB) and occasional white matter iron rich glia (blue arrowheads, Fe). (For interpretation of the references to colour in this figure legend, the reader is referred to the web version of this article.)

On MRI, atypical signal was largely localized in the ATC, consistent with the patient’s clinical presentation of svPPA with bvFTD features. (Gorno-Tempini et al., 2011) ATC showed substantial atrophy and a hypointense “band” in the upper layers of the cortex, along with mild diffuse dot-like hypointense speckling across the entire cortical depth with loss of distinct cortical GM lamination seen in the healthy control sample. A thin band of juxtacortical WM also showed overall hypointense signal compared to relative hyperintense signal in the focal area of relative deep WM to this cortical region. This regionally hyperintense signal in WM was accompanied with diffuse hypointense striations on MRI (Fig. 3, solid blue arrowheads).

Histopathological analysis of ATC showed a high density of TDP-43 positive long- dystrophic neurites that was most prominent in upper layers and associated with relatively greater superficial cortical layer neuronal loss and vacuolization. TDP-43 pathology was largely absent in WM, similar to previous reports for FTLD-TDP type C. (Mackenzie and Neumann, 2020) The remainder of regions had mild or absent TDP-43 and neurodegeneration.

Corresponding to the observed upper layer hypointense band and diffuse cortical speckling seen on MRI, iron staining in ATC revealed a high density of staining in cortical layer II, consisting of a mixture of dot-like stippling pattern of bead-like dystrophic appearing processes and additional prominent reactivity of cellular processes surrounding small blood vessels (Fig. 3, solid blue arrowheads; Fig. 9, open blue arrowheads). Examination of adjacent sections for glial markers suggested that these iron-rich structures correspond to diffuse punctate and tortuous IBA-1-positive dystrophic microglial processes (Fig. 9, solid yellow stars) and GFAP-positive astrocytic processes enveloping small vessels (Fig. 9, open blue arrowheads), respectively. Moreover, there were moderate amounts of iron-rich cellular structures resembling rod-shaped or ameboid microglial morphologies in layers III-VI and occasionally in juxtacortical WM that corresponded to activated microglia visualized by IBA-1 immunostaining (Fig. 9, solid yellow arrowheads). Histologically, WM staining in ATC showed scant intracortical myelin and mild relative loss of adjacent WM compared to control tissue.

#### 3.4.2 Patient #9 (Replication Cohort)

This replication patient had FTLD-TDP type C and antemortem features of bvFTD along with svPPA. Based on our findings in patient #3, we focused our analysis on the ATC of patient #9. On MRI we found focal degeneration in ATC with diffuse dot-like speckling and upper layer hypointense band with reduced signal in adjacent WM, similar to patient #3. Histopathological examination of ATC confirmed the correlation of radiographic findings to upper-layer iron-rich gliosis in astrocytic processes near small vessels and dot-like stippling throughout the cortex to a mixture of hypertrophic and dystrophic iron-reactive microglial processes in GM. There was loss of myelin in adjacent WM and less common iron-rich glia.

### 3.5 Patient #4 (Discovery Cohort) – FTLD-TDP type a Neuropathology, Behavioral-Variant Frontotemporal dementia syndrome

**Fig. 4:**
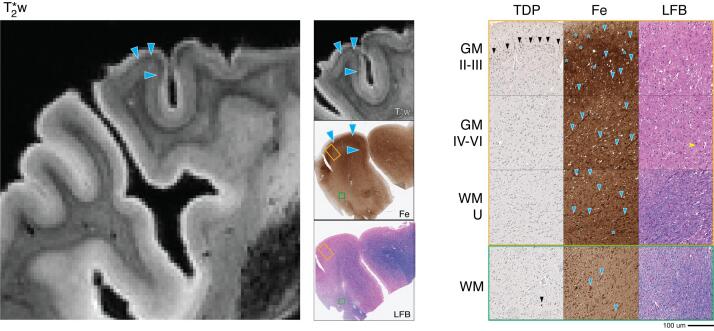
FTLD-TDP type A (patient #4) lateral orbitofrontal MRI and pathology. *Left:* T2*w MRI of lateral orbitofrontal region (including sampled BA 47). *Center:* low-magnification (1x) view of tissue sample stained for iron (Fe) and myelin (LFB), with corresponding T2*w MRI slice. *Right:* high-magnification (20x) tissue sample stained for TDP-43 pathology (TDP), Fe, and LFB (scale bar = 100 µm). Rows depict view in upper cortical layers (GM II-III), deep cortical layers (GM IV-VI), directly adjacent white matter enriched for cortical U-fibers (WM-U) and relatively deeper white matter (WM) from boxes outlined in low magnification view (orange box = cortical layers, green box = deep WM). There is prominent TDP-43 pathology largely restricted to upper cortical layers (black arrow heads, TDP) along with severe neuronal loss and vacuolization (LFB). T2*w MRI shows a hypointense upper layer band and diffuse speckling (blue arrow heads, T2*w) which correlated with iron deposits resembling astrocyte processes surrounding capillaries (blue arrow heads, Fe) and iron-rich glia (blue stars, Fe). There were sparse deep fibers in the deeper cortical layers (yellow notched arrow head, LFB). Adjacent white matter signal was heterogenous on MRI, corresponding to mild reduction in relative deep white matter myelin (LFB) and occasional white matter deposits and gliosis (blue arrowheads, Fe). (For interpretation of the references to colour in this figure legend, the reader is referred to the web version of this article.)

On MRI, both ATC and OFC showed atypical diffuse dot-like speckling throughout the cortex, including upper layers. There was focal hyperintense signal in WM adjacent to ATC with a gradient of darker signal in juxtacortical WM and some scant WM striations of hypointense signal. Similar to the TDP C patients (patients #3 and #9), we found an upper-cortical hypointense band on MRI, here in a lateral region of OFC (BA 47) (Fig. 4, solid blue arrowheads).

In ATC and OFC, histopathology showed highest TDP-43 pathology in cytoplasmic inclusions and diffuse neurites, accompanied by severe upper layer neuronal loss and vacuolization in regions sampled, consistent with known pathological and regional patterns of FTLD-TDP A. (Mackenzie and Neumann, 2020, Mackenzie et al., 2011) There were also mild TDP-43 inclusions in WM oligodendrocytes in these regions, while the remainder of regions sampled had scant or absent TDP-43 and minimal or no neurodegeneration of GM or WM.

The MRI-guided lateral orbitofrontal cortex (LOFC) sample showed a similar pattern on histopathology, with more pronounced iron-rich astrocytic processes enveloping small vessels in layers II-III and overall darker appearance of the neuropil, particularly in upper layers (Fig. 4, solid blue arrowheads), similar to patients #3 and #9 with FTLD-TDP C. Iron-reactive pathology corresponded to a high level of GFAP-reactive astrocytes highlighting small capillaries (Fig. 9, open blue arrowheads) and beaded dystrophic IBA-1 reactive microglial processes (Fig. 9, solid yellow stars). Intracortical myelin was mildly reduced, as was adjacent WM. In the standard sampled OFC and ATC there was a similar pattern with less prominent upper-layer iron-rich gliosis and ATC had more prominent iron-positive hypertrophic ameboid microglia.

### 3.6 4R-Predominant globular glial tauopathy Neuropathology, Non-Fluent/Agrammatic PPA and corticobasal syndromes

#### 3.6.1 Patient #5 (Discovery Cohort)

**Fig. 5:**
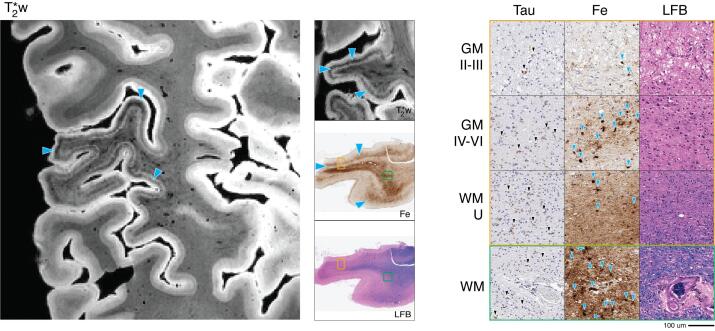
FTLD-Tau GGT 4R tauopathy (patient #5) primary motor MRI and pathology. *Left:* T2*w MRI of the inferior aspect of the primary motor region (including sampled BA 4). *Center:* low-magnification (1x) view of tissue sample stained for iron (Fe) and myelin (LFB), with corresponding T2*w MRI slice. *Right:* high-magnification (20x) tissue sample stained for tau pathology (Tau), Fe, and LFB (scale bar = 100 µm). Rows depict view in upper cortical layers (GM II-III), deep cortical layers (GM IV-VI), directly adjacent white matter enriched for cortical U-fibers (WM-U) and relatively deeper white matter (WM) from boxes outlined in low magnification view (orange box = cortical layers, green box = deep WM). There is widespread tau pathology (black arrowheads, Tau) across cortical layers and in white matter along with severe neuronal loss and vacuolization across layers (LFB). T2*w MRI shows a large irregular hypointense band (blue arrow heads, T2*w) across mid to lower cortical layers. This coincided on histopathology with clusters iron deposits resembling hypertrophic appearing microglia (blue arrow heads, Fe). There was no apparent MRI signal corresponding to the band of Baillarger and adjacent white matter was striated with large hypointensities near blood vessels. This pattern corresponded on pathology to an absence of intracortical white matter and severe myelin loss in adjacent white matter, along with large clusters of iron deposits resembling glia surrounding larger blood vessels (blue arrow heads, Fe). (For interpretation of the references to colour in this figure legend, the reader is referred to the web version of this article.)

MRI showed prominent abnormal signal in primary motor cortex, which showed frank irregular hypointense “smudges” within the deep layers of cortical laminae. Adjacent WM showed large irregular hypointense striations with vessel-like shapes. These findings were most pronounced in the inferior aspect of the motor cortex near the areas involved with motor speech, matching the patient’s clinical presentation of naPPA and later emerging features of corticobasal syndrome (CBS). (Gorno-Tempini et al., 2011) We performed MRI-guided sampling of an additional inferior area of motor cortex with most prominent imaging features (Fig. 5, solid blue arrowheads).

This patient displayed 4R-predominant pathology in tau-positive “globular” oligodendrocytic tau inclusions in GM and WM that was most prominent in the corticospinal tract as well as astrocytic tau pathology, tangles and threads in all layers of cortical GM, consistent with globular glial tauopathy type II. (Ahmed et al., 2013) Tau pathology and resultant neurodegeneration were severe and most prominent in primary motor and inferior motor regions, while milder in IPFC and OFC and negligible in the primary somatosensory region. There was also mild to moderate amount of co-morbid diffuse amyloid beta plaque (stage A2, C0- Table 1) most concentrated in OFC and IPFC with rare iron-reactivity resembling plaques for these regions only.

Corresponding to irregular hypointense signal on MRI, iron stain of primary and inferior motor regions revealed a central band (approximately layers III-V) of dense, highly reactive iron-positive clusters resembling ameboid and hypertrophic microglia (Fig. 5, solid blue arrowheads; Fig. 9, solid yellow arrowheads). There were also lighter iron-reactive deposits corresponding to astrocyte morphologies in mid-cortical layers and in sub-pial areas of layer I. Adjacent tissue stained for glial markers confirmed the presence of large ameboid-type microglia in mid to deeper layers of GM and throughout WM, as well as a high density of weakly GFAP-positive reactive protoplasmic astrocytes (Fig. 9, solid blue arrowheads) and many with some dystrophic features (Fig. 9, solid blue stars) throughout GM layers with a relative depletion of fibrous astrocytes in WM compared to the healthy control sample. Myelin stain showed minimal existing intracortical myelin and moderate loss of WM in juxtacortical and relative deep areas of adjacent WM. Rare diffuse amyloid plaques that did not react to iron stain were noted in primary and inferior motor cortex. These plaques were spatially distinct from the area of novel iron-rich glial pathology in this sample (Supplementary Fig. 2). Iron staining of subjacent WM showed similar patches of high density of iron-positive microglia, often in focal clusters surrounding large blood vessels. Vessel walls appeared thickened with enlarged surrounding space and mild extracellular hemosiderin deposits but no evidence of amyloid angiopathy in these large vessels in relatively deeper WM (Supplementary Fig. 2).

#### 3.6.2 Patient #10 (Replication Cohort)

Based on our findings in patient #5, we focused our analysis on the motor cortex of patient #10 with non-fluent PPA and later emerging CBS motor features corresponding to underlying GGT 4R tauopathy. MRI showed a similar deep hypointense irregular banding pattern in mid-to-deep cortical layers and in adjacent WM, particularly near large vessels. Focused histopathological validation in primary motor cortex found very high levels of iron-rich ameboid microglia most prominent in deeper cortical layers and adjacent WM with severe neuronal and WM degeneration, similar to patient #5.

### 3.7 Patient #6 – 4R-Predominant PSP tauopathy Neuropathology, Steele-Richardson progressive supranuclear palsy syndrome

**Fig. 6:**
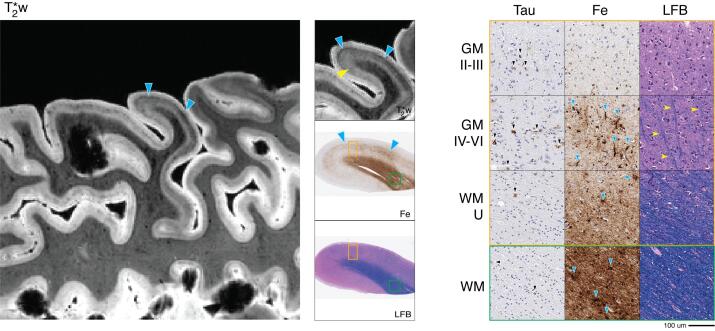
FTLD-Tau PSP 4R tauopathy (patient #6) primary motor MRI and pathology. *Left:* T2*w MRI of the primary motor region (including sampled BA 4). *Center:* low-magnification (1x) view of tissue sample stained for iron (Fe) and myelin (LFB), with corresponding T2*w MRI slice. *Right:* high-magnification (20x) tissue sample stained for tau pathology (Tau), Fe, and LFB (scale bar = 100 µm). Rows depict view in upper cortical layers (GM II-III), deep cortical layers (GM IV-VI), directly adjacent white matter enriched for cortical U-fibers (WM-U) and relatively deeper white matter (WM) from boxes outlined in low magnification view (orange box = cortical layers, green box = deep WM). There is widespread tau pathology (black arrowheads, Tau) across cortical layers and in white matter along with moderate neuronal loss across layers (LFB). MRI showed a large irregular hypointense band across mid-to-lower cortical layers (blue arrowheads, T2*w); on histopathology this correlated to clusters of iron deposits resembling hypertrophic appearing microglia (blue arrowheads, Fe). There was some preserved shading on MRI (yellow notched arrowhead, T2*w) that was partially obscured by the pathological iron signal; this pattern corresponded to the preservation of deep cortical layer myelin (yellow notched arrowheads, LFB). Adjacent white matter showed patchy hypointensities, which corresponded on pathology to the mild reduction in myelin (LFB) and additional large clusters of iron deposits resembling glia (blue arrowheads, Fe). (For interpretation of the references to colour in this figure legend, the reader is referred to the web version of this article.)

MRI results in our standardized sampling regions identified a distinct pathological feature in primary motor cortex, which was notable for a large irregular hypointense “smudge” in the middle layers of cortex with some irregular extension of mild hypointensity into the subjacent WM relative to nearby gyri (Fig. 6, solid blue arrowheads), similar to GGT. This irregular hypointense band partially obscured the signal from intracortical WM. Finally, there was large-sized speckling across the cortical layers in ATC and IPFC which were reminiscent of that seen in the ADNC patient.

Histopathology was typical for PSP, with tau-positive tangles and tufted astrocytes throughout cortical layers and tau-positive threads and coiled bodies in adjacent WM that was most severe in the primary motor region. (Kovacs et al., 2020) This patient had moderate amyloid-beta plaque co-pathology (Table 1) and a subgroup of amyloid-plaques were iron-reactive in ATC and IPFC, correlating with our observation on MRI of “large-size speckling” in the ATC and IPFC in this patient. In contrast, amyloid-beta plaque co-pathology was mild in primary motor cortex, and spatially distinct from the band of unique iron-positive hypertrophic microglia in this sample (Supplementary Fig. 2) described below.

Corresponding to the deep-layer hypointensity on MRI, iron stain showed clustered deposits resembling activated microglia in layers III-V and adjacent WM (Fig. 6, solid blue arrowheads; Fig. 9, solid yellow arrowheads), similar to GGT patient samples. These clusters were occasionally associated with large blood vessels in GM and WM. There were less distinct astrocyte morphologies seen on iron stain (Fig. 9, solid blue arrowheads), and adjacent tissue stained for GFAP showed mild reactivity in mid-layers and similar findings to controls in WM (Fig. 9, solid blue arrowheads). Myelin histology revealed preserved deep layer cortical myelin and adjacent WM.

### 3.8 Patient #7 – 3R-Predominant Pick’s disease tauopathy Neuropathology, Behavioral-Variant Frontotemporal dementia syndrome

**Fig. 7:**
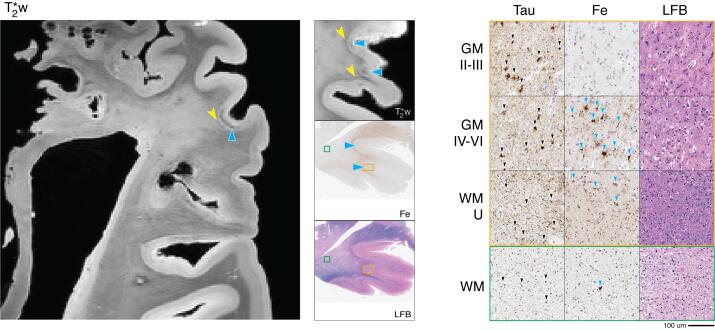
FTLD-Tau Pick’s disease 3R tauopathy (patient #7) dorsolateral prefrontal MRI and pathology. *Left:* T2*w MRI of a dorsolateral prefrontal region (including sampled BA 9). *Center:* low-magnification (1x) view of tissue sample stained for iron (Fe) and myelin (LFB), with corresponding T2*w MRI slice. *Right:* high-magnification (20x) tissue sample stained for tau pathology (Tau), Fe, and LFB (scale bar = 100 µm). Rows depict view in upper cortical layers (GM II-III), deep cortical layers (GM IV-VI), directly adjacent white matter enriched for cortical U-fibers (WM-U) and relatively deeper white matter (WM) from boxes outlined in low magnification view (orange box = cortical layers, green box = deep WM). There is widespread tau pathology (black arrow heads, Tau) across cortical layers and in white matter along with severe neuronal loss across layers (LFB). MRI shows a large irregular hypointense band (blue arrowheads, T2*) in mid-to-lower cortical layers; this coincided on pathology with clusters of iron-rich hypertrophic appearing microglia and astrocytic profiles (blue arrowheads, Fe). There were also clusters of iron rich glia in juxtacortical WM (blue arrowheads, Fe) associated on MRI with relative hypointensity of juxtacortical WM (yellow notched arrowheads, T2*w) compared to the hyperintense deeper WM. (For interpretation of the references to colour in this figure legend, the reader is referred to the web version of this article.)

MRI identified OFC and IPFC as abnormal among our standard sampling. In both of these regions, and diffusely thought the frontal cortices, there was overall WM hyperintensity relative to posterior healthy-appearing WM associated with primary motor and somatosensory regions, with the effect most pronounced in OFC. In frontal association regions there was an atypical lack of contrast from deep and upper cortical layers as seen in the healthy control brain. We located an additional focal area in the dorsolateral midfrontal cortex (DLPFC, BA9) with mid-to-deep cortical hypointense band which we sampled for additional histopathologic analysis (Fig. 7, solid blue arrowheads).

Histopathologic examination identified tau-positive Pick bodies in neurons and tau-positive astrocytes in a ramified morphology typical for tau immunostaining (Kovacs et al., 2016) throughout the cortical layers and associated with severe neuronal loss typical for Pick’s disease in the OFC, IPFC, and DLPFC. (Irwin et al., 2016) This was accompanied by severe tau pathology in the form of WM threads and oligodendrocytes in these regions. In contrast, tau pathology and neurodegeneration were minimal in primary motor and sensory cortex.

Iron staining in DLPFC found prominent clusters of iron-positive activated microglia (Fig. 9, solid yellow arrowheads) and dystrophic processes (Fig. 9, solid yellow stars) in mid to deep GM layers (layers III-VI) and juxtacortical WM and less prominent iron-positive protoplasmic astrocytes in deep GM (Fig. 9, solid blue arrowheads). There were also scattered reactive iron-positive microglia in deeper adjacent WM. Interestingly, glial staining in adjacent tissue revealed a relative depletion of microglia in DLPFC with clusters of microglia with ameboid profiles (Fig. 9, solid yellow arrowheads) in a similar spatial distribution to iron stain and severe widespread GFAP reactivity in activated astrocytes and their processes in GM and WM (Fig. 9, solid blue arrowheads). In contrast, OFC and IFC, which had similar tau pathology and neurodegeneration to DLPFC, had only scant iron deposits resembling glia. Thus, the observed hyperintensity of cortex on MRI appeared to derive from both the lack of cortical myelin and rarity of iron-rich glia. Finally, iron staining in OFC, IPFC and DLFPC also reflected the absence of cortical myelin and reduced adjacent WM integrity correlating with MRI findings of indistinguishable cortical lamination and hyper intense signal in adjacent WM in these regions (Fig. 7).

### 3.9 Histopathologic replication cohort

To help confirm the generalizability of results, we performed iron-staining from archived tissue in our brain bank with uniform overnight fixation from FTLD brains without *ex vivo* MRI. We selected regions from our brain bank based on our MRI-guided discovery analyses above.

In these histopathologic replication analyses, we found laminar patterns of iron-rich gliosis in our standard sampled regions from previous autopsies, similar to those detected in our discovery cohort. We noted more prominent upper layer iron reactivity largely in astrocytic processes enveloping small vessels and dystrophic and hypertrophic microglia in FTLD-TDPA and an additional FTLD-TDPC sample (Table 2). In FTLD-Tau with PSP we found mild to moderate deep activated microglia reactive to iron, largely concentrated in deep layers while FTLD-Tau with PiD had variable hypertrophic and dystrophic microglia along with less common astrocytic morphologies, most prominent in deeper cortical layers.

**Table 2:** Histopathological Replication Cohort and Data.

**Pathology Group**	FTLD-tau PSP	FTLD-tau PiD	FTLD-TDP A	FTLD-TDP C
N (Male/Female)	9 (5/4)	10 (9/1)	10 (4/6)	1 (0/1)
Brain Weight (g)	1204.4 (110.5)	1072 (126.9)	905.6 (109.3)	1101
PMI (hours)	11.5 (6.5)	22 (15.7)	9.9 (6.1)	18
Age at Death (years)	76.6 (7.2)	65.4 (5.7)	72.1 (10.9)	61
Disease duration (years)	6.4 (3.2)	10.7 (5.1)	7 (3.0)	6
ABC score	A0B0C0 = 4 A0B1C0 = 2 A0B2C0 = 3	A0B0C0 = 7 A0B1C0 = 1 A0BNAC0 = 2*	A0B0C0 = 8 A0B1C0 = 1 A0B2C0 = 1	A0B0C0 = 1
Region	Primary Motor	Mid-frontal	Orbitofrontal	Anterior Temporal
Astrocyte processes- perivascular GM Upper Layer	0 (0,0)	0.75 (0.125, 1)	2 (2.75)	3
Astrocyte processes- perivascular GM Deep Layer	0 (0,0)	1 (0.5, 1)	1 (1,2)	2
Ameboid Microglia GM Upper Layer	0 (0, 0.5)	0.5 (0.5, 0.875)	1 (1,1)	0.5
Ameboid Microglia GM Deep Layer	2 (1,3)	2 (1,2)	0.75 (0.5, 1)	0

Cells denote frequency for categorical data, Mean (Standard Deviation) for normally-distributed demographics, and Median (Interquartile Range) for ordinal pathology scores of iron-rich glia morphologies in archived brain bank tissue.

* = 2 Picks disease tauopathies had no available Braak stage due to severe tauopathy.

### 3.10 Contralateral hemisphere replication of histopathologic iron findings

FTLD pathology is often asymmetrically distributed across hemispheres (Irwin et al., 2018), so we examined iron reactivity in a paired sample obtained fresh at autopsy from the contralateral hemisphere available in six of our patients with *ex vivo* MRI. These contralateral samples produced similar histopathologic results to our MRI-guided analysis in the ipsilateral scanned hemisphere (Supplementary Fig. 4).

## 4 Discussion

We used an integrated, *ex vivo* MRI and histopathology approach to study diverse FTLD clinical and pathological subtypes, and contrasted these with ADNC and healthy brain samples. This enabled novel findings of distinct upper layer hypointense bands and diffuse speckling on T2*w MRI which corresponded to both iron-rich astrocytic processes surrounding small blood vessels and dystrophic (as opposed to hypertrophic) patterns of microglia, respectively in FTLD-TDP. In contrast, these patterns were not observed in FTLD-Tau and instead we found large irregular hypointense signal in deep cortical layers and adjacent WM on T2*w MRI that correlated with iron-rich hypertrophic microglia. Moreover, there was loss of normal cortical myelination patterns and hyperintense signal in adjacent WM corresponding to severe WM degeneration particularly evident in the 3R tauopathy PiD brain.

We then replicated these findings in a second cohort with both *ex vivo* MRI and histopathology, as well as in a larger histopathological replication cohort in archived FTLD-Tau and FTLD-TDP tissue, demonstrating reproducibility of our results. Thus, our findings suggest potential divergent mechanisms of neuroinflammation and resultant neurodegeneration between specific pathological forms of FTLD that may be detectible during life. We also demonstrate the utility of *ex vivo* MRI to guide histopathologic work needed to study these novel patterns of gliosis outside of traditional histopathological sampling optimized for AD. (Montine et al., 2012)

### 4.1 Focal iron-positive gliosis within the cortical laminae and adjacent white matter in FTLD proteinopathies

**Fig. 8:**
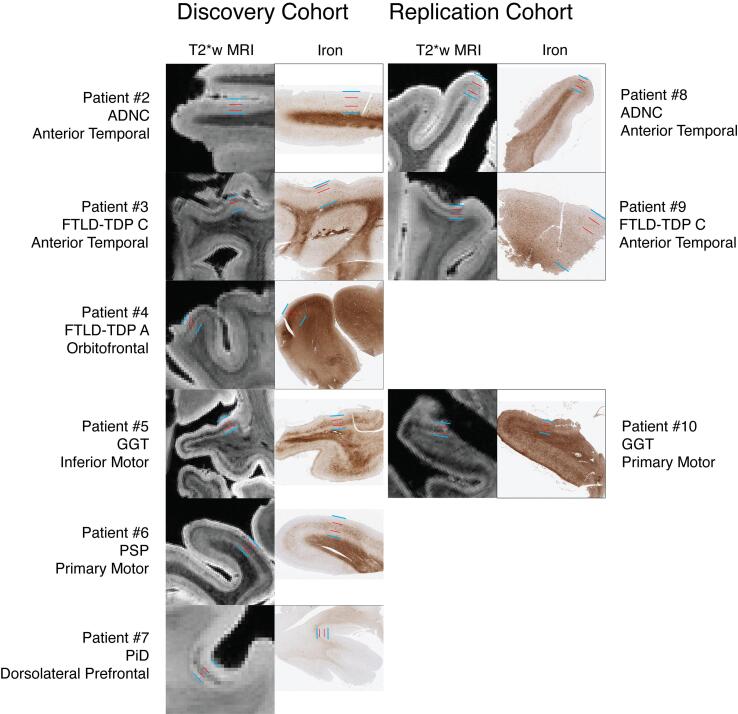
Laminar pathology in T2*w MRI and iron stains across discovery and replication cohorts. T2*w MRI and photomicrographs of iron-stained sections across our discovery (left column) and replication (right column) cohorts. MR and iron-stain images are manually aligned, except in patients #7 and #9, where the displayed MRI slices are perpendicular to the histology slices (although showing the same pathologic foci), to better-demonstrate the laminar features. Blue calipers depict the upper and lower extent of the cortex, while red calipers depict the upper and lower extent of the pathologic iron inclusions visualized on both T2*w MRI and iron-stained sections. Regions for each disease type were chosen by either standardized or image-guided sampling in the discovery cohort. These same regions were then selected in the replication cohort before inspection of the MRI, demonstrating the reproducibility of our findings of iron-rich pathology in ADNC, FTLD-TDP, and FTLD-tau cases. (For interpretation of the references to colour in this figure legend, the reader is referred to the web version of this article.)

**Fig. 9:**
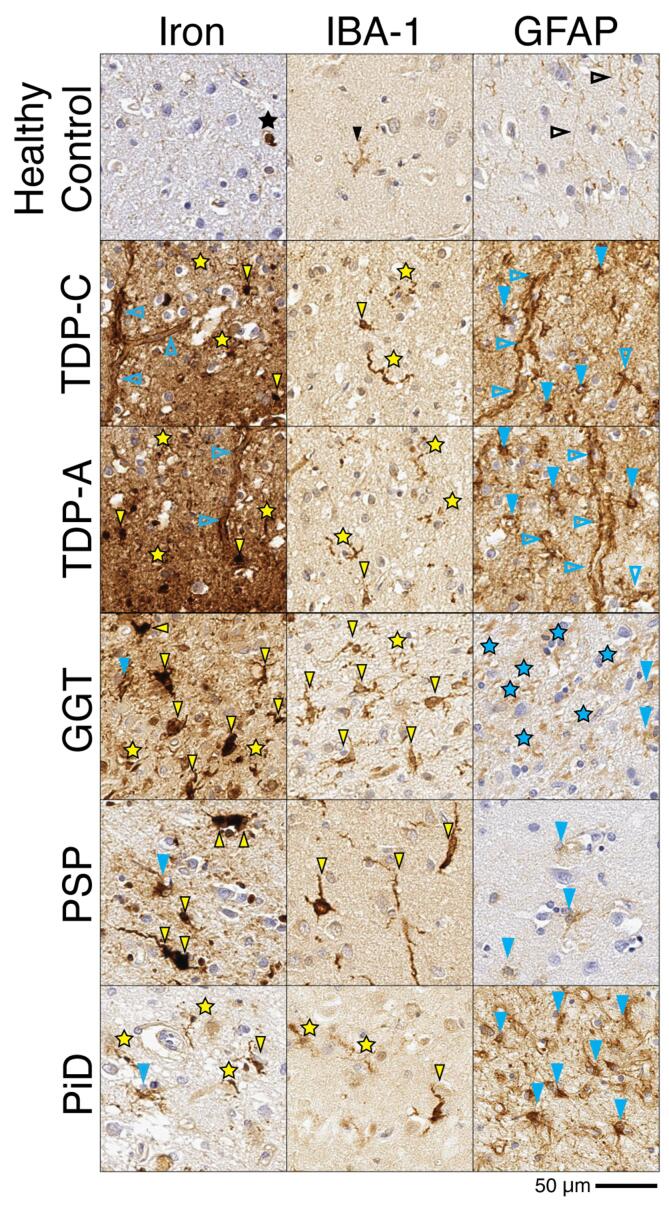
Patterns of iron-rich glia in FTLD-TDP and FTLD-Tau groups. Photomicrographs depict representative images from iron-stained sections with abnormal T2* signal ex vivo in FTLD samples and semi-adjacent sections immunostained for microglia (IBA-1) and activated astrocytes (GFAP). The healthy control had iron-reactivity largely restricted to oligodendrocytes (black star). Glial immunostaining in healthy control shows mild amounts of microglia in ramified non-reactive morphology (solid black arrowhead) and mild diffuse GFAP + fibrous astrocytic processes in upper cortical layers (open black arrow heads). The FTLD-TDP patients (TDPA and TDPC) had shared features of relative upper layer (layers II-III) iron in processes surrounding capillaries (open blue arrowheads) as well as diffuse speckling of the neuropil (yellow stars) corresponding to a high level of GFAP reactive astroglial processes (open blue arrowheads) and dystrophic processes (yellow stars) of IBA-1 reactive microglia (solid yellow arrowheads), respectively. In contrast, FTLD-Tau patients had prominent clusters of hypertrophic “ameboid” and “rod-like” appearing iron-rich profiles (solid yellow arrowheads) that were most conspicuous in middle to deeper cortical layers and corresponded to IBA-1 reactive microglia in hypertrophic morphologies (solid yellow arrowheads). The GGT sample also showed dystrophic processes (yellow stars) on iron-stain and IBA-1, with prominent fragmented dystrophic GFAP + processes (blue stars) throughout. In contrast, PSP showed moderate level of astrogliosis (open blue arrowhead) that were less similar in morphology to adjacent iron-stained cellular structures. The Pick’s disease (PiD) 3R tauopathy had similar, but relatively isolated, clusters of hypertrophic iron-rich microglia (solid yellow arrowheads) in deep grey matter and adjacent juxtacortical white matter that corresponded to activated IBA-1 morphologies and degenerating processes (yellow stars). There were also occasional iron-rich morphologies similar to GFAP-reactive activated astrocytes (solid blue arrowheads) which were more prominent in PID than in the 4R tauopathies, but in contrast to FTLD-TDP, were seldom iron-rich. Scale bar = 50 µm. (For interpretation of the references to colour in this figure legend, the reader is referred to the web version of this article.)

In FTLD-TDP samples we found an irregular upper-layer hypointense band within the cortex on MRI (Fig. 8), corresponding to iron deposits and GFAP-positive astrocytic processes enveloping small blood vessels. Additionally, hypointense speckling in the cortex of diseased regions was observed on MRI throughout cortical layers that correlated on histopathology with dystrophic microglial processes labelled by IBA-1 (Fig. 3, Fig. 4, Fig. 9). These findings are similar to previous reports of iron-rich gliosis in motor cortex of ALS with TDP-43 proteinopathy, but contrast with the laminar distribution described in ALS as mid-to-deep layers. (Kwan et al., 2012, Oba et al., 1993) Our study did not include ALS or TDP-B, both of which have greater relative deep layer pathology compared to TDP-A and -C, (Mackenzie et al., 2011) which may contribute to this discrepancy. Nonetheless, these patterns were distinct from those seen in FTLD-Tau patients in our cohort.

On MRI, the 4R tauopathy cases (i.e., GGT and PSP, patients #5, #6, and #10) had prominent mid-to-deep layer irregular hypointense “smudges” in GM (Fig. 8), and vessel-shaped hypointensities in nearby WM. On pathology, these corresponded to clusters of iron-rich activated microglia often surrounding large vessels (Fig. 5, Fig. 6, Fig. 9). While the gross anatomical location of a high burden of pathology in primary motor cortex in these tauopathies31, (Ahmed et al., 2013) partially overlaps with regional patterns of pathology in ALS, (Kwan et al., 2012, Brettschneider et al., 2013) the MRI and pathological features of these FTLD-Tau cases are distinct in the addition of prominent iron-rich gliosis in adjacent WM. Indeed, a previous report of GGT histology also finds similar pattern of iron-rich gliosis in deep GM and adjacent WM, (Hasegawa et al., 2018) and we found this pattern in primary motor cortex of PSP cases in our histopathological replication cohort (Table 2).

In PiD, the 3R tauopathy (patient 7), there were shared features with 4R tauopathies of focal, deep-layer hypointensity on MRI (Fig. 8), occurring in this case in DLPFC. On pathology, this was associated with a mixture of activated astrocytes and dystrophic microglia processes that were also evident in juxtacortical WM (Fig. 6, Fig. 9). Moreover, we found similar iron-reactivity in nearby mid-frontal cortex sampling in our archived brain tissue (Table 2).

Despite similar clinical symptomatology, our FTLD samples had distinct MRI and pathological features from our ADNC patient. Several studies in amnestic AD syndrome using *ex vivo* MRI and pathology have described non-homogenous appearance of cortex with hypointense speckling in layers III-V that correlates with varying amounts of iron-rich plaque and associated microglia on histology. (Benveniste et al., 1999, Bulk et al., 2018, Zeineh et al., 2015) Similar to the previous studies, we observed this mid-layer speckling pattern in our ADNC subject with antemortem bvFTD diagnosis, which was associated on histology with a subset of neuritic plaques and activated microglia forming morphologies and distributions concordant with iron deposits in mid cortical layers (Fig. 3). Interestingly, the ATC, a region implicated in bvFTD, (Josephs et al., 2009) was an area of high density of iron deposits and corresponding amyloid-beta plaque pathology in this patient. There is considerable heterogeneity of clinical presentations associated with ADNC and recently a dysexecutive subtype has been proposed. (Alladi et al., 2007, Ossenkoppele et al., 2015, Townley et al., 2020) Our cases had a prominent social disorder consistent with bvFTD (Table 1) and thus would not meet these new proposed clinical criteria for dysexecutive ADNC. (Townley et al., 2020)

### 4.2 Histological analysis of iron-positive glia suggests disparate mechanisms of iron homeostasis and inflammation in AD and FTLD subtypes

Our histological analysis found only a subset of activated glia appear to sequester iron (Fig. 9). Alterations of iron homeostasis have been linked to neurodegeneration as deficits in autophagy may lead to lysosome-mediated release of ferric iron from ferritin in degenerating cells (reviewed in detail elsewhere(Biasiotto et al., 2016, Rao and Adlard, 2018, Ward et al., 2014)). Glial activation may also contribute to neurodegeneration in AD, FTLD, and related disorders via non-cell autonomous mechanisms altering the microenvironment of the brain from chronic neuroinflammation. (Heppner et al., 2015, Ransohoff, 2016)

The large iron-rich ameboid morphologies seen in areas of high-pathology in 4R tauopathies suggest the pro-inflammatory state may involve iron sequestration from phagocytosis of cellular debris. (Boche et al., 2013) In particular, the iron-stained features consistent with ameboid microglia in WM were uniquely localized near larger blood vessels in our 4R tauopathy samples. Indeed, ameboid activated microglia have been described in 4R tauopathies, (Hasegawa et al., 2018, Ishizawa and Dickson, 2001) and similar findings of a large burden of perivascular phagocytic microglia were reported in a hereditary tauopathy. (Bellucci et al., 2011) While we saw similar iron-rich ameboid morphology in TDP samples, these were less common and more focally only in the ATC of FTLD-TDP subtype A patient #4.

In contrast to the ameboid form, we also noted cases where iron deposits had a beaded appearance that morphologically resemble senescent microglia. (Streit et al., 2020, Streit et al., 2004) This was particularly evident in FTLD-TDP (patients #3, #4, and #9), and focally in deep layers of the DLPFC of the PiD sample (patient #7). These findings align with previous reports of prominent upper layer microglial activity in forms of FTLD-TDP, (Lant et al., 2014, Taipa et al., 2017) while PiD shows a variable but more uniform distribution across layers. (Cooper et al., 1996, Lant et al., 2014)

More generally, in our FTLD-TDP samples we found localized iron deposits and corresponding microglia in focal areas of cortical degeneration, in contrast to the large-vessel patterns seen in large areas of WM in 4R tauopathies. Indeed, activated microglia have been associated with focal areas of cortical degeneration in FTLD-TDP suggesting an association of WM microglia with Wallerian degeneration of axons from neuronal loss. (Ohm et al., 2019)

GFAP-reactive astrocytes are normally found in healthy white matter in a fibrous morphology, (Beach et al., 1989) as seen in our healthy control sample. However in our GGT and PiD sample (patients #5, #10, and #7) , there was a conspicuous absence of these fibrous astrocytes in WM and instead there was fragmentation of GFAP-positive processes in GGT (Fig. 9), similar to previous reports of astrocytic degeneration in FTLD-Tau. (Hsu et al., 2018, Martinac et al., 2001) FTLD-Tau has prominent tau pathology within astrocytes and this mechanism could contribute to the patterns observed here.

In contrast, FTLD-TDP inclusions are seldom localized within astrocytes (Lee et al., 2017) and we did not observe degeneration of astrocytes in these samples. Instead, in FTLD-TDP we found prominent GFAP-reactive astrocytic morphologies in diseased regions, highlighted by iron deposits resembling astrocytic processes surrounding small vessels in upper cortical layers (Fig. 3, Fig. 4, Fig. 9). Similar to previous reports, (Kersaitis et al., 2004, Schofield, 2003, Cooper et al., 1996) we also observed high levels of GFAP-positive gliosis in PiD across all cortical layers but, in contrast to FTLD-TDP, these cellular structures were largely not visualized by iron staining, suggesting a potential unique process of astrocyte-mediated iron dysregulation in TDP-43 proteinopathies. This is consistent with the likely contribution of astrocytes to iron homeostasis via active transport uptake of peripheral iron. (Pelizzoni et al., 2013)

### 4.3 Differential depletion of healthy myelin across FTLD, ADNC, and control

Healthy myelin, as observed in our healthy control tissue, has distinct laminar distribution across cortical regions (i.e. myeloarchitecture) with most prominent intracortical myelin found in deeper layers (Nieuwenhuys et al., 2015). Moreover, previous work has found various myelination patterns in other primary and secondary associative cortices (Fracasso et al., 2016) that we largely recapitulate here (Supplementary Fig. 1). In our FTLD samples, we found novel evidence of striking deviation from these established patterns of myelination in healthy cortex and adjacent WM.

The most obvious loss of intracortical myelin was seen in our PiD sample, appearing on MRI as widespread homogenous hyperintense signal in frontal association cortex, corresponding histologically to obliteration of deep-layer myelin and severe neuron loss throughout all layers (Fig. 7). A similar but more focal pattern of intracortical myelin loss was seen in the GGT patient in primary motor cortex (Fig. 5) while the band of Baillarger in the PSP sample (Fig. 6) was relatively preserved. Heterogeneity observed among tauopathies could be attributed to tau isoform type (3R vs 4R), individual differences in rate of progression, disease duration, and/or the relative minimal distribution of PSP tauopathy in cortical regions compared to brainstem and subcortical regions not examined here. (Kovacs et al., 2020) In FTLD-TDP samples, there was also evidence for degeneration of intracortical WM that correlated with severe neuronal loss and was most prominent in upper layers II-III (Fig. 3, Fig. 4). Thus, neuronal loss and WM pathology may contribute to loss of myelination in our samples.

This differed from the pattern of cortical lamination in our atypical ADNC patient. While largely preserved on histology, cortical lamination was obscured in many locations on MRI by the non-homogeneous hypointense speckling associated with iron deposits resembling and spatially correlated with a subset of neuritic plaques and associated glia (Fig. 3). One previous study found a subset of advanced ADNC patients, largely with young age of onset, to have an abnormal mid-layer band pattern on T2*w MRI corresponding to increased size and disorganization of intracortical myelin. (Bulk et al., 2018, van Duijn et al., 2017) We did not observe this pattern in our ADNC patient samples, which, had a relative older age at onset (over age 65; see Table 1) which could account for this discrepancy.

### 4.4 Limitations and future work

The strong correlation between *ex vivo* T2*w MRI contrast and the presence of iron in our pathology analyses suggests that iron-based contrast in reactive/degenerating glia and healthy myelin is the largest, most consistent effect across our samples. Other sources of T2*w have been suggested. For example, diamagnetism of tau and beta amyloid inclusions has been quantified *in vitro* and in transgenic mice, finding that tau protein inclusions were significantly diagmagnetic at echo times in the range used in our study, with beta amyloid showing a smaller effect. (Gong et al., 2019) In our sample this does not appear to produce significant T2*w contrast separate from iron, as exemplified in our PiD sample (patient #7), where OFC and IPFC had substantial tau inclusions but minimal iron, and was generally homogenous and hyperintense on MRI (Supplementary Tables 1–4). Therefore, other MRI contrasts, such as magnetization transfer, may be more sensitive to other histopathological substrates in FTLD spectrum and can be compared to T2*w MRI to further elucidate patterns of degeneration in distinct proteinopathies. Additionally, while we have used a single-echo image for a first report of qualitative histopathological correlation in FTLD, future work could likely extract additional information by employing all acquired echoes to quantitatively estimate R2* or local off-resonance frequency to further enhance sensitivity to iron and myelin. (Fukunaga et al., 2010, Hametner et al., 2018)

There may be interhemispheric differences in microscopic pathology within individual patients which could influence results. (Irwin et al., 2018) In the subset of patients with focal iron-rich gliosis in standard sampled regions (i.e. primary motor cortex, anterior temporal lobe) we had tissue obtained fresh at the time of autopsy from the contralateral hemisphere and these showed similar patterns of iron-rich gliosis as in our imaged hemispheres (Supplementary Fig. 4).

Methodological confounds could also contribute to our findings, but we performed careful histological procedures using optimized methods for iron-stain, (Meguro et al., 2007, van Duijn et al., 2013) run in triplicate and confirmed with comparison to ferritin IHC (Supplementary Fig. 3). It is important to consider pre-analytical factors that could influence our results. There was variability in fixation times across samples in our discovery and replication cohorts, particularly for PiD patient #7 (∼21 months). However, all other samples were in formalin for<6 months prior to scanning and so iron leakage or other tissue degradation is unlikely contributory as this has been observed only in much longer fixation times beyond five years. (Gellein et al., 2008) Postmortem interval prior to fixation can also affect tissue in regards to phosphorylation state of proteins, enzyme denaturation and RNA integrity. (Matsuo et al., 1994) However, our samples had relatively short post-mortem interval (<24 h, Table 1), well within the range of robust immunohistochemical analysis of brain tissue. (Blair et al., 2016, Lesnikova et al., 2018)

While the diversity of our samples’ clinical and pathological features has enabled us to detect unique and frank iron-rich features on joint MRI/histopathology, it is important to acknowledge the limitation of our small sample size in a pathologically heterogeneous disorder. We mitigated this limitation through use of a replication cohort of additional samples with joint *ex vivo* MRI and histopathology, as well as second histological replication cohort examined for iron-reactivity in our archived brain tissues without *ex vivo* imaging. However, further replication using joint *ex vivo* imaging and histology are needed to help establish foundational work for neuroimaging biomarker development in FTLD. Our findings raise questions regarding the role of inflammation and oxidative stress, but these are dynamic processes with likely varying rates of progression across regions and individual patients. Therefore, we cannot entirely rule out an alternative possibility that all pathological forms of FTLD undergo a similar mechanism of inflammation and iron-overload, captured here at different stages in individual patient samples. Nonetheless, our findings of distinct laminar iron-rich features and corresponding gliosis in areas with high disease burden may also be evident earlier in the disease during life.

Moreover, our work here establishes the basis for future work in larger datasets exploring these focal features. Additionally, our imaging methods, applied in a larger dataset, will enable more quantitative comparison of MRI and digital histology data within and between groups to further inform these complex mechanistic issues.

## 5 Conclusion

Our results indicate that FTLD with underlying TDP-43 or tau proteinopathy subtypes have distinct mesoscopic signatures within the cortical laminae and neighboring white matter that produce frank features on *ex vivo* T2*w MRI. On histopathology, these features correspond with distinct patterns of microscopic iron-rich pathology, strongly indicative of disparate disease processes amongst FTLD subtypes characterized by prominent upper layer iron-rich astrocytes surrounding small vessels and diffuse iron-rich dystrophic microglia in TDP-43 proteinopathies, while tauopathies were characterized by hypertrophic microglia in deep layers and adjacent WM. Joint MRI/histopathology studies in FTLD offer a unique method for localizing and studying these focal features — both their spatial distribution across the cortex and their microscopic features.

In addition, previous work has demonstrated that *in vivo* 7 T MRI can be sensitized to the intra-cortical pathologic iron content due to ADNC, (Gong et al., 2019, Kenkhuis et al., 2019, van Rooden et al., 2014) and ALS, (Acosta-Cabronero et al., 2018, Kwan et al., 2012), although not with sufficient resolution to clearly resolve the layer-specific effects we describe here. However, recent work in a small number of healthy subjects has shown the feasibility of increasing *in vivo* cortical laminar resolution of 7 T MRI to 250 µm in focal regions, enabling detection of layer-specific myleoarchitecture within small patches of cortex. (Balasubramanian et al., 2021) We therefore expect that the novel iron-rich pathology described here may form a basis for future *in vivo* imaging biomarkers to localize disease processes, distinguish TDP-43 and tau proteinopathies, and further stratify FTLD subtypes.

## CRediT authorship contribution statement

**M. Dylan Tisdall:** Conceptualization, Methodology, Software, Formal analysis, Investigation, Resources, Data curation, Writing – original draft, Visualization. **Daniel T. Ohm:** Investigation, Writing – review & editing. **Rebecca Lobrovich:** Investigation. **Sandhitsu R. Das:** Software, Writing – review & editing. **Gabor Mizsei:** Resources. **Karthik Prabhakaran:** Investigation. **Ranjit Ittyerah:** Investigation. **Sydney Lim:** Investigation. **Corey T. McMillan:** Resources, Writing – review & editing. **David A. Wolk:** Resources, Writing – review & editing. **James Gee:** Supervision. **John Q. Trojanowski:** Resources, Writing – review & editing. **Edward B. Lee:** Resources, Writing – review & editing. **John A. Detre:** Resources, Writing – review & editing. **Paul Yushkevich:** Resources, Writing – review & editing. **Murray Grossman:** Resources, Writing – review & editing, Supervision, Funding acquisition. **David J. Irwin:** Conceptualization, Methodology, Formal analysis, Investigation, Resources, Data curation, Writing – original draft, Visualization, Supervision, Funding acquisition.

## Declaration of Competing Interest

The authors declare that they have no known competing financial interests or personal relationships that could have appeared to influence the work reported in this paper.
