# Supplementary material for: Ex vivo MRI and histopathology detect novel iron-rich cortical inflammation in frontotemporal lobar degeneration with tau versus TDP-43 pathology
Source: Neuroimage Clin. 2021 Dec 14;33:102913. doi: 10.1016/j.nicl.2021.102913 (PMC8715243; doi:10.1016/j.nicl.2021.102913)
Supplement: Supplementary data 1 [file mmc1.docx]

**Ex vivo MRI and histopathology detect novel iron-rich cortical inflammation in frontotemporal lobar degeneration with tau versus TDP-43 pathology**

**SUPPLEMENTARY MATERIALS**

**Supplementary Table 1. Neuropathological and MRI Hypointensity Ratings.**

| **Patient** | **Region** | **Layer** | **NL** | **Inclusion** | **Amyloid** | **Fe** | **Ferritin** | **IBA1** | **GFAP** | **MRI** |
| --- | --- | --- | --- | --- | --- | --- | --- | --- | --- | --- |
| 1  Healthy Control | Orbitofrontal | Upper GM | 0 | 0 | 0 | 0 | 0.5 | 1 | 1 | 0 |
|  |  | Deep GM | 0 | 0 | 0 | 0 | 0.5 | 1 | 0 | 0 |
|  |  | WM-U | N/A | 0 | 0 | 0 | 0 | 1 | 3 | - |
|  |  | WM Deep | N/A | 0 | 0 | 0 | 0 | 1 | 3 | 0 |
|  | Inferior Prefrontal | Upper GM | 0 | 0 | 0 | 0.5 | 0.5 | 0.5 | 1 | 0 |
|  |  | Deep GM | 0 | 0 | 0 | 0.5 | 0.5 | 0.5 | 0 | 0 |
|  |  | WM-U | N/A | 0 | 0 | 0 | 0 | 1 | 3 | - |
|  |  | WM Deep | N/A | 0 | 0 | 0 | 0 | 1 | 3 | 0 |
|  | Primary Motor | Upper GM | 0 | 0 | 0.5 | 0 | 0.5 | 0.5 | 0 | 0 |
|  |  | Deep GM | 0 | 0 | 0 | 0 | 0.5 | 0.5 | 0 | 0 |
|  |  | WM-U | N/A | 0 | 0 | 0 | 0.5 | 0.5 | 3 | - |
|  |  | WM Deep | N/A | 0 | 0 | 0 | 0.5 | 0.5 | 3 | 0 |
|  | Primary Sensory | Upper GM | 0 | 0 | 0.5 | 0 | 0.5 | 0.5 | 1 | 0 |
|  |  | Deep GM | 0 | 0 | 0.5 | 0 | 0.5 | 0.5 | 0 | 0 |
|  |  | WM-U | N/A | 0 | 0 | 0 | 0 | 0.5 | 3 | - |
|  |  | WM Deep | N/A | 0 | 0 | 0 | 0 | 0.5 | 3 | 0 |
| 2  ADNC | Anterior Temporal | Upper GM | 2 | 1 | 3 | 2 | 3 | 3 | 3 | 2 |
|  |  | Deep GM | 2 | 1 | 3 | 3 | 3 | 3 | 2 | 3 |
|  |  | WM-U | N/A |  | 0.5 | 2 | 2 | 1 | 3 | - |
|  |  | WM Deep | N/A |  | 0.5 | 1 | 2 | 1 | 2 | 0 |
|  | Orbitofrontal | Upper GM | 1 | 3 | 3 | 2 | 2 | 2 | 2 | 1 |
|  |  | Deep GM | 1 | 3 | 3 | 3 | 2 | 2 | 2 | 2 |
|  |  | WM-U | N/A | 0 | 0 | 1 | 0.5 | 2 | 2 | - |
|  |  | WM Deep | N/A | 0 | 0 | 0 | 0.5 | 2 | 2 | - |
|  | Inferior Prefrontal | Upper GM | 2 | 3 | 3 | 1 | 2 | 1 | 2 | 1 |
|  |  | Deep GM | 1 | 3 | 3 | 2 | 2 | 2 | 2 | 2 |
|  |  | WM-U | N/A | 0.5 | 0.5 | 0.5 | 2 | 1 | 2 | - |
|  |  | WM Deep | N/A | 0.5 | 0.5 | 0 | 2 | 1 | 2 | 0 |
|  | Primary Motor | Upper GM | 0 | 2 | 2 | 1 | 2 | 1 | 1 | 0 |
|  |  | Deep GM | 0 | 2 | 2 | 2 | 2 | 1 | 1 | 0 |
|  |  | WM-U | N/A | 0.5 | 0 | 1 | 0 | 1 | 2 | - |
|  |  | WM Deep | N/A | 0.5 | 0 | 0 | 0 | 1 | 2 | 0 |
|  | Primary Sensory | Upper GM | 1 | 3 | 3 | 0 | 2 | 2 | 2 | 0 |
|  |  | Deep GM | 1 | 3 | 3 | 1 | 2 | 2 | 2 | 0 |
|  |  | WM-U | N/A | 0 | 0 | 0 | 1 | 2 | 2 | - |
|  |  | WM Deep | N/A | 0 | 0 | 0 | 1 | 2 | 2 | 0 |
| 3  TDPC | Anterior Temporal | Upper GM | 3 | 3 | 0 | 3 | 2 | 3 | 3 | 3 |
|  |  | Deep GM | 2 | 3 | 0 | 2 | 1 | 3 | 3 | 3 |
|  |  | WM-U | N/A | 0.5 | 0 | 1 | 1 | 3 | 3 | - |
|  |  | WM Deep | N/A | 0.5 | 0 | 1 | 1 | 3 | 3 | 2 |
|  | Orbitofrontal | Upper GM | 2 | 2 | 0 | 1 | 2 | 3 | 2 | 0 |
|  |  | Deep GM | 1 | 1 | 0 | 1 | 2 | 3 | 0 | 0 |
|  |  | WM-U | N/A | 0.5 | 0 | 0.5 | 0.5 | 3 | 2 | - |
|  |  | WM Deep | N/A | 0.5 | 0 | 0.5 | 0.5 | 3 | 1 | 0 |
|  | Inferior Prefrontal | Upper GM | 1 | 1 | 0 | 0.5 | 1 | 1 | 1 | 0 |
|  |  | Deep GM | 0 | 0.5 | 0.5 | 0.5 | 1 | 0.5 | 0 | 0 |
|  |  | WM-U | N/A | 0 | 0 | 0 | 1 | 1 | 2 | - |
|  |  | WM Deep | N/A | 0 | 0 | 0 | 1 | 0.5 | 2 | 0 |
|  | Primary Motor | Upper GM | 0 | 0.5 | 0 | 0.5 | 1 | 1 | 1 | 0 |
|  |  | Deep GM | 0 | 0 | 0 | 0 | 1 | 1 | 0 | 0 |
|  |  | WM-U | N/A | 0 | 0 | 0 | 1 | 1 | 2 | - |
|  |  | WM Deep | N/A | 0 | 0 | 0 | 1 | 1 | 2 | 0 |
|  | Primary Sensory | Upper GM | 0 | 0 | 0 | 0.5 | 1 | 1 | 1 | 0 |
|  |  | Deep GM | 0 | 0 | 0 | 0.5 | 1 | 1 | 0 | 0 |
|  |  | WM-U | N/A | 0 | 0 | 0 | 0.5 | 1 | 2 | - |
|  |  | WM Deep | N/A | 0 | 0 | 0 | 0.5 | 1 | 2 | 0 |
| 4  TDPA | Anterior Temporal | Upper GM | 2 | 2 | 0 | 1 | 3 | 3 | 2 | 2 |
|  |  | Deep GM | 1 | 1 | 0 | 2 | 2 | 2 | 1 | 2 |
|  |  | WM-U | N/A | 0.5 | 0 | 1 | 1 | 1 | 2 | - |
|  |  | WM Deep | N/A | 0.5 | 0 | 1 | 1 | 1 | 1 | 2 |
|  | Orbitofrontal | Upper GM | 3 | 3 | 0 | 2 | 3 | 3 | 3 | 2 |
|  |  | Deep GM | 1 | 0.5 | 0 | 2 | 2 | 2 | 2 | 3 |
|  |  | WM-U | N/A | 0.5 | 0 | 1 | 1 | 1 | 2 | - |
|  |  | WM Deep | N/A | 0.5 | 0 | 1 | 1 | 1 | 0.5 | 2 |
|  | Lateral  Orbitofrontal (MRI) | Upper GM | 2 | 3 | 0 | 3 | 3 | 2 | 3 | 3 |
|  |  | Deep GM | 0 | 1 | 0 | 2 | 2 | 1 | 2 | 1 |
|  |  | WM-U | N/A | 1 | 0 | 1 | 1 | 0.5 | 3 | - |
|  |  | WM Deep | N/A | 1 | 0 | 1 | 1 | 0.5 | 3 | 2 |
|  | Inferior Prefrontal | Upper GM | 1 | 1 | 0 | 0.5 | 2 | 1 | 1 | 0 |
|  |  | Deep GM | 0 | 0 | 0 | 0 | 1 | 0.5 | 0 | 0 |
|  |  | WM-U | N/A | 0 | 0 | 0 | 1 | 0.5 | 2 | - |
|  |  | WM Deep | N/A | 0 | 0 | 0 | 1 | 0.5 | 2 | 0 |
|  | Primary Motor | Upper GM | 1 | 1 | 0 | 1 | 2 | 2 | 1 | 0 |
|  |  | Deep GM | 0 | 0 | 0 | 1 | 1 | 1 | 0 | 0 |
|  |  | WM-U | N/A | 0 | 0 | 0 | 1 | 1 | 2 | - |
|  |  | WM Deep | N/A | 0 | 0 | 0 | 1 | 1 | 2 | 0 |
|  | Primary Sensory | Upper GM | 0 | 0 | 0 | 0.5 | 1 | 1 | 0.5 | 0 |
|  |  | Deep GM | 0 | 0 | 0 | 0.5 | 0.5 | 1 | 0 | 0 |
|  |  | WM-U | N/A | 0 | 0 | 0 | 0 | 1 | 2 | - |
|  |  | WM Deep | N/A | 0 | 0 | 0 | 0 | 1 | 2 | 0 |
| 5  GGT | Orbitofrontal | Upper GM | 0 | 0.5 | 2 | 0 | 1 | 1 | 1 | 0 |
|  |  | Deep GM | 0 | 0.5 | 2 | 0.5 | 1 | 1 | 1 | 0 |
|  |  | WM-U | N/A | 0.5 | 0 | 0 | 1 | 2 | 2 | - |
|  |  | WM Deep | N/A | 0.5 | 0 | 0 | 1 | 2 | 2 | 0 |
|  | Inferior Motor  (MRI) | Upper GM | 3 | 3 | 0.5 | 1 | 3 | 3 | 3 | 1 |
|  |  | Deep GM | 3 | 3 | 0.5 | 3 | 3 | 3 | 3 | 3 |
|  |  | WM-U | N/A | 3 | 0 | 2 | 3 | 3 | 1 | - |
|  |  | WM Deep | N/A | 3 | 0 | 2 | 3 | 3 | 1 | 3 |
|  | Inferior Prefrontal | Upper GM | 1 | 1 | 2 | 1 | 2 | 2 | 2 | 0 |
|  |  | Deep GM | 1 | 1 | 2 | 2 | 2 | 2 | 2 | 0 |
|  |  | WM-U | N/A | 1 | 0 | 0.5 | 1 | 3 | 2 | - |
|  |  | WM Deep | N/A | 1 | 0.5 | 0.5 | 1 | 3 | 2 | 0 |
|  | Primary Motor | Upper GM | 3 | 2 | 0.5 | 1 | 3 | 3 | 3 | 1 |
|  |  | Deep GM | 3 | 3 | 0.5 | 3 | 3 | 3 | 3 | 3 |
|  |  | WM-U | N/A | 3 | 0 | 2 | 3 | 3 | 2 | - |
|  |  | WM Deep | N/A | 3 | 0 | 2 | 3 | 3 | 1 | 3 |
|  | Primary Sensory | Upper GM | 0 | 0.5 | 0.5 | 0 | 2 | 2 | 2 | 0 |
|  |  | Deep GM | 0 | 0.5 | 0.5 | 0 | 2 | 2 | 2 | 0 |
|  |  | WM-U | N/A | 1 | 0 | 0 | 2 | 3 | 2 | - |
|  |  | WM Deep | N/A | 1 | 0 | 0 | 2 | 3 | 2 | 0 |
| 6  PSP | Anterior Temporal | Upper GM | 0 | 1 | 2 | 2 | 2 | 2 | 2 | 0 |
|  |  | Deep GM | 0 | 1 | 2 | 2 | 2 | 2 | 1 | 0 |
|  |  | WM-U | N/A | 0.5 | 0 | 0.5 | 2 | 3 | 2 | - |
|  |  | WM Deep | N/A | 0.5 | 0 | 0.5 | 2 | 3 | 2 | 0 |
|  | Orbitofrontal | Upper GM | 0 | 0.5 | 3 | 0.5 | 3 | 2 | 1 | 0 |
|  |  | Deep GM | 0 | 0.5 | 3 | 1 | 3 | 2 | 0 | 0 |
|  |  | WM-U | N/A | 0.5 | 0 | 1 | 2 | 3 | 2 | - |
|  |  | WM Deep | N/A | 0.5 | 0 | 1 | 2 | 3 | 2 | 0 |
|  | Inferior Prefrontal | Upper GM | 1 | 1 | 2 | 1 | 3 | 2 | 2 | 2 |
|  |  | Deep GM | 1 | 2 | 2 | 2 | 3 | 2 | 1 | 3 |
|  |  | WM-U | N/A | 1 | 0 | 1 | 2 | 3 | 2 | - |
|  |  | WM Deep | N/A | 1 | 0 | 1 | 2 | 3 | 2 | 1 |
|  | Primary Motor | Upper GM | 1 | 2 | 0.5 | 1 | 3 | 3 | 3 | 0 |
|  |  | Deep GM | 1 | 3 | 0.5 | 3 | 3 | 2 | 3 | 3 |
|  |  | WM-U | N/A | 2 | 0 | 2 | 2 | 3 | 3 | - |
|  |  | WM Deep | N/A | 2 | 0 | 2 | 2 | 3 | 3 | 1 |
|  | Primary Sensory | Upper GM | 0 | 1 | 1 | 0.5 | 2 | 1 | 1 | 0 |
|  |  | Deep GM | 0 | 1 | 1 | 1 | 2 | 1 | 1 | 0 |
|  |  | WM-U | N/A | 1 | 0 | 0 | 2 | 1 | 2 | - |
|  |  | WM Deep | N/A | 1 | 0 | 0 | 2 | 2 | 2 | 0 |

| 7  PiD | Orbitofrontal | Upper GM | 3 | 3 | 0 | 0.5 | 2 | 1 | 2 | 0 |
| --- | --- | --- | --- | --- | --- | --- | --- | --- | --- | --- |
|  |  | Deep GM | 3 | 3 | 0 | 1 | 2 | 1 | 1 | 0 |
|  |  | WM-U | N/A | 1 | 0 | 2 | 1 | 1 | 2 | - |
|  |  | WM Deep | N/A | 1 | 0 | 1 | 1 | 1 | 2 | 0 |
|  | Dorsolateral Prefrontal (MRI) | Upper GM | 3 | 3 | 0 | 0.5 | 3 | 3 | 3 | 0 |
|  |  | Deep GM | 3 | 3 | 0 | 2 | 3 | 3 | 3 | 2 |
|  |  | WM-U | N/A | 3 | 0 | 2 | 2 | 1 | 3 | - |
|  |  | WM Deep | N/A | 3 | 0 | 1 | 2 | 1 | 3 | 2 |
|  | Inferior Prefrontal | Upper GM | 3 | 3 | 0 | 0 | 2 | 1 | 2 | 0 |
|  |  | Deep GM | 3 | 3 | 0 | 2 | 2 | 2 | 1 | 0 |
|  |  | WM-U | N/A | 1 | 0 | 2 | 1 | 1 | 2 | - |
|  |  | WM Deep | N/A | 1 | 0 | 1 | 1 | 1 | 2 | 0 |
|  | Primary Motor | Upper GM | 0 | 0.5 | 0 | 0 | 2 | 1 | 0 | 0 |
|  |  | Deep GM | 0 | 0.5 | 0 | 0.5 | 1 | 1 | 0 | 0 |
|  |  | WM-U | N/A | 0 | 0 | 0 | 1 | 1 | 1 | - |
|  |  | WM Deep | N/A | 0 | 0 | 0 | 1 | 1 | 1 | 0 |
|  | Primary Sensory | Upper GM | 0 | 0.5 | 0 | 0 | 0.5 | 0.5 | 0.5 | 0 |
|  |  | Deep GM | 0 | 0.5 | 0.5 | 0.5 | 0.5 | 0.5 | 0 | 0 |
|  |  | WM-U | N/A | 0.5 | 0 | 0 | 0 | 1 | 1 | - |
|  |  | WM Deep | N/A | 0.5 | 0 | 0 | 0 | 1 | 1 | 0 |
| 8  ADNC | Anterior Temporal | Upper GM | 1 | 3 | 3 | 1 | 2 | 3 | 3 | 1 |
|  |  | Deep GM | 1 | 3 | 3 | 3 | 2 | 3 | 3 | 3 |
|  |  | WM-U | N/A | 1 | 0 | 0.5 | 1 | 3 | 2 | - |
|  |  | WM Deep | N/A | 0 | 0 | 0.5 | 1 | 3 | 2 | 1 |
| 9  TDPC | Anterior Temporal | Upper GM | 3 | 3 | 0 | 2 | 2 | 3 | 3 | 3 |
|  |  | Deep GM | 1 | 3 | 0 | 1 | 1 | 1 | 3 | 2 |
|  |  | WM-U | N/A | 0.5 | 0 | 1 | 1 | 1 | 3 | - |
|  |  | WM Deep | N/A | 0 | 0 | 2 | 1 | 2 | 3 | 2 |
| 10  GGT | Primary Motor | Upper GM | 2 | 2 | 0 | 2 | 2 | 3 | 3 | 1 |
|  |  | Deep GM | 3 | 3 | 0 | 3 | 3 | 3 | 3 | 3 |
|  |  | WM-U | N/A | 3 | 0 | 3 | 2 | 2 | 2 | - |
|  |  | WM Deep | N/A | 3 | 0 | 3 | 2 | 1 | 2 | 3 |

Table depicts ordinal ratings in each patient sample region on standard ordinal rating scale (0=none, 0.5=rare, 1=mild, 2=moderate, 3=severe). NL= neuronal loss from LFB stained slides, Inclusion= proteinopathy severity (tau for FTLD-Tau, AD and HC; TDP-43 for FTLD-TDP), Amyloid= amyloid plaque score, Fe= score for glial pathology detected by Perl’s stain for iron with DAB enhancement, Ferritin= = score for glial pathology detected by ferritin immunostain, IBA-1= activated microglial score from IBA-1 immunostain, GFAP= activated astrocyte score for GFAP immunostain, MRI = abnormal hypointensity score for T2*w MRI, ADNC= Alzheimer’s disease neuropathologic change, TDPA= FTLD-TDP subtype A, TDPC= FTLD-TDP subtype C, GGT= FTLD-tau with globular glial tauopathy, PSP= FTLD-tau with progressive supranuclear palsy, PiD = FTLD-Tau with Pick’s disease, upper GM = grey matter in upper cortical layers II-III, deep GM = GM in deep cortical layers IV-VI , WM-U = juxtacortical white matter enriched for U-fibers, WM Deep = relative deep WM, N/A = not applicable as no neuron loss to assess in WM layers.

**Supplementary Table 2. Histopathological ratings of white matter integrity.**

| **Patient** | **Cortical Region** | **Layer** | **WM LFB** | **WM Iron** |
| --- | --- | --- | --- | --- |
| 1  Healthy Control | Orbitofrontal | Deep GM | 2 | 2 |
|  |  | WM-U | 3 | 3 |
|  |  | WM Deep | 3 | 3 |
|  | Inferior Prefrontal | Deep GM | 2 | 2 |
|  |  | WM-U | 3 | 3 |
|  |  | WM Deep | 3 | 3 |
|  | Primary Motor | Deep GM | 3 | 3 |
|  |  | WM-U | 3 | 3 |
|  |  | WM Deep | 3 | 3 |
|  | Primary Sensory | Deep GM | 3 | 3 |
|  |  | WM-U | 3 | 3 |
|  |  | WM Deep | 3 | 3 |
| 2  ADNC | Anterior Temporal | Deep GM | 0.5 | 0.5 |
|  |  | WM-U | 3 | 3 |
|  |  | WM Deep | 3 | 3 |
|  | Orbitofrontal | Deep GM | 1 | 1 |
|  |  | WM-U | 3 | 3 |
|  |  | WM Deep | 3 | 3 |
|  | Inferior Prefrontal | Deep GM | 2 | 2 |
|  |  | WM-U | 3 | 3 |
|  |  | WM Deep | 3 | 3 |
|  | Primary Motor | Deep GM | 3 | 2 |
|  |  | WM-U | 3 | 2 |
|  |  | WM Deep | 3 | 2 |
|  | Primary Sensory | Deep GM | 3 | 2 |
|  |  | WM-U | 3 | 3 |
|  |  | WM Deep | 3 | 3 |
| 3  TDPC | Anterior Temporal | Deep GM | 0.5 | 0.5 |
|  |  | WM-U | 1 | 3 |
|  |  | WM Deep | 3 | 3 |
|  | Orbitofrontal | Deep GM | 1 | 0.5 |
|  |  | WM-U | 3 | 3 |
|  |  | WM Deep | 3 | 3 |
|  | Inferior Prefrontal | Deep GM | 2 | 2 |
|  |  | WM-U | 3 | 3 |
|  |  | WM Deep | 3 | 3 |
|  | Primary Motor | Deep GM | 3 | 3 |
|  |  | WM-U | 3 | 3 |
|  |  | WM Deep | 3 | 3 |
|  | Primary Sensory | Deep GM | 3 | 3 |
|  |  | WM-U | 3 | 3 |
|  |  | WM Deep | 3 | 3 |
| 4  TDPA | Anterior Temporal | Deep GM | 0.5 | 0.5 |
|  |  | WM-U | 2 | 3 |
|  |  | WM Deep | 3 | 3 |
|  | Orbitofrontal | Deep GM | 0.5 | 0.5 |
|  |  | WM-U | 2 | 2 |
|  |  | WM Deep | 1 | 1 |
|  | Lateral Orbitofrontal (MRI) | Deep GM | 1 | 1 |
|  |  | WM-U | 2 | 3 |
|  |  | WM Deep | 2 | 3 |
|  | Inferior Prefrontal | Deep GM | 2 | 2 |
|  |  | WM-U | 3 | 2 |
|  |  | WM Deep | 3 | 2 |
|  | Primary Motor | Deep GM | 3 | 3 |
|  |  | WM-U | 3 | 2 |
|  |  | WM Deep | 3 | 2 |
|  | Primary Sensory | Deep GM | 3 | 2 |
|  |  | WM-U | 3 | 2 |
|  |  | WM Deep | 3 | 2 |

| 5  GGT | Orbitofrontal | Deep GM | 1 | 1 |
| --- | --- | --- | --- | --- |
|  |  | WM-U | 3 | 2 |
|  |  | WM Deep | 3 | 2 |
|  | Inferior Motor (MRI) | Deep GM | 0 | 0 |
|  |  | WM-U | 2 | 2 |
|  |  | WM Deep | 2 | 2 |
|  | Inferior Prefrontal | Deep GM | 1 | 1 |
|  |  | WM-U | 3 | 2 |
|  |  | WM Deep | 3 | 2 |
|  | Primary Motor | Deep GM | 0.5 | 0 |
|  |  | WM-U | 2 | 2 |
|  |  | WM Deep | 1 | 2 |
|  | Primary Sensory | Deep GM | 3 | 2 |
|  |  | WM-U | 3 | 2 |
|  |  | WM Deep | 3 | 2 |
| 6  PSP | Anterior Temporal | Deep GM | 1 | 1 |
|  |  | WM-U | 3 | 3 |
|  |  | WM Deep | 3 | 3 |
|  | Orbitofrontal | Deep GM | 2 | 1 |
|  |  | WM-U | 3 | 3 |
|  |  | WM Deep | 3 | 3 |
|  | Inferior Prefrontal | Deep GM | 2 | 2 |
|  |  | WM-U | 3 | 3 |
|  |  | WM Deep | 3 | 3 |
|  | Primary Motor | Deep GM | 3 | 2 |
|  |  | WM-U | 3 | 3 |
|  |  | WM Deep | 3 | 3 |
|  | Primary Sensory | Deep GM | 3 | 3 |
|  |  | WM-U | 3 | 3 |
|  |  | WM Deep | 3 | 3 |
| 7  PiD | Orbitofrontal | Deep GM | 0 | 0 |
|  |  | WM-U | 1 | 1 |
|  |  | WM Deep | 1 | 1 |
|  | Dorsolateral Prefrontal (MRI) | Deep GM | 0 | 0 |
|  |  | WM-U | 2 | 1 |
|  |  | WM Deep | 1 | 0.5 |
|  | Inferior Prefrontal | Deep GM | 0.5 | 0.5 |
|  |  | WM-U | 1 | 1 |
|  |  | WM Deep | 1 | 1 |
|  | Primary Motor | Deep GM | 3 | 1 |
|  |  | WM-U | 2 | 2 |
|  |  | WM Deep | 2 | 2 |
|  | Primary Sensory | Deep GM | 1 | 1 |
|  |  | WM-U | 3 | 2 |
|  |  | WM Deep | 3 | 2 |
| 8  ADNC | Anterior Temporal | Deep GM | 2 | 2 |
|  |  | WM-U | 2 | 3 |
|  |  | WM Deep | 2 | 3 |
| 9  TDPC | Anterior Temporal | Deep GM | 0.5 | 0.5 |
|  |  | WM-U | 1 | 2 |
|  |  | WM Deep | 1 | 2 |
| 10  GGT | Primary Motor | Deep GM | 0.5 | 0.5 |
|  |  | WM-U | 1 | 2 |
|  |  | WM Deep | 1 | 2 |

Table depicts ordinal ratings of white matter in each patient sample region on standard ordinal rating scale (0=severe myelin degeneration with no discernable fibers, 0.5= severe myelin degeneration with rare isolated fibers, 1=moderate myelin degeneration, 2= mild myelin degeneration, 3=healthy appearing myelin without degeneration). WM LFB = white matter detected by luxol-fast blue myelin stain, WM Iron = white matter detected by Perl’s stain for iron with DAB enhancement, ADNC= Alzheimer’s disease neuropathologic change, TDPA= FTLD-TDP subtype A, TDPC= FTLD-TDP subtype C, GGT= FTLD-tau with globular glial tauopathy, PSP= FTLD-tau with progressive supranuclear palsy, PiD = FTLD-Tau with Pick’s disease, deep GM= WM in deep cortical layers (band of Baillarger), WM-U= juxtacortical white matter enriched for U-fibers, WM deep= relative deep WM.

| **Region** | **Feature** | **Pt#1**  **Healthy Control** | **Pt#2**  **ADNC** | **Pt#3**  **TDPC** | **Pt#4**  **TDPA** | **Pt#5**  **GGT** | **Pt#6**  **PSP** | **Pt#7**  **PiD** |
| --- | --- | --- | --- | --- | --- | --- | --- | --- |
| Anterior Temporal | WM Subj./Overall | - | -1 | 0 | 0 | 0 | 0 | - |
|  | WM UF/Subj. | - | -1 | -1 | -2 | 0 | 0 | - |
|  | Upper/Lower Layers | - | 0 | 0 | 0 | 0 | 0 | - |
|  | BoB/GM | - | -1 | -1 | 0 | -1 | -2 | - |
|  | GM/Subj. WM | - | +2 | +2 | +2 | +2 | +2 | - |
|  | Atyp. Features | - | DS++ | DS, UB, WS | DS, MB, WS |  | DS++ | - |
| Orbitofrontal | WM Subj./Overall | 0 | 0 | 0 | 0 | 0 | 0 | +2 |
|  | WM UF/Subj. | -2 | -1 | -1 | -2 | -1 | 0 | -2 |
|  | Upper/Lower Layers | 0 | 0 | +1 | 0 | 0 | 0 | 0 |
|  | BoB/GM | 0 | 0 | 0 | -1 | 0 | -1 | 0 |
|  | GM/Subj. WM | +2 | +2 | +2 | +1 | +2 | +2 | 0 |
|  | Atyp. Features |  | DS |  | DS |  |  |  |
| Inferior Prefrontal | WM Subj./Overall | 0 | 0 | 0 | 0 | 0 | 0 | +1 |
|  | WM UF/Subj. | -2 | 0 | -1 | -1 | 0 | 0 | -1 |
|  | Upper/Lower Layers | +2 | +1 | +2 | +2 | +2 | 0 | 0 |
|  | BoB/GM | -1 | -1 | -2 | -1 | -1 | -1 | -1 |
|  | GM/Subj. WM | +2 | +2 | +2 | +2 | +2 | +2 | +2 |
|  | Atyp. Features |  | DS |  |  |  | DS |  |
| Primary Motor | WM Subj./Overall | 0 | 0 | 0 | 0 | -2 | -1 | 0 |
|  | WM UF/Subj. | -1 | -1 | -1 | 0 | 0 | 0 | 0 |
|  | Upper/Lower Layers | +2 | +1 | +2 | +2 | +2 | +2 | +2 |
|  | BoB/GM | -2 | -1 | -2 | -1 | a | a | -1 |
|  | GM/Subj. WM | +2 | +2 | +2 | +2 | +2 | +2 | +2 |
|  | Atyp. Features |  |  |  |  | MB++, WS | MB,  WS |  |
| Primary Sensory | WM Subj./Overall | 0 | 0 | 0 | 0 | 0 | 0 | 0 |
|  | WM UF/Subj. | -1 | -1 | -1 | 0 | -1 | 0 | 0 |
|  | Upper/Lower Layers | +2 | +1 | +2 | +2 | +1 | +2 | +2 |
|  | BoB/GM | -2 | -1 | -2 | -1 | -2 | -2 | -1 |
|  | GM/Subj. WM | +2 | +2 | +2 | +2 | +2 | +2 | +2 |
|  | Atyp. Features |  |  |  |  |  |  |  |

**Supplementary Table 3. T2*w MRI contrast in standardized sampling regions**

ADNC= Alzheimer’s disease neuropathologic change, TDPA= FTLD-TDP subtype A, TDPC= FTLD-TDP subtype C, GGT= FTLD-tau with globular glial tauopathy, PSP= FTLD-tau with progressive supranuclear palsy, PiD = FTLD-Tau with Pick’s disease; DS = diffuse speckling, MB = mid-cortical hypointense band, WS = striated white matter; a = BoB was completely occluded by mid-cortical hypointense band through entire gyrus.

**Supplementary Table 4. T2*w MRI contrast in MRI-guided sampling regions**

| **Feature** | **Pt#4**  **TDPA**  **Lateral Orbitofrontal** | **Pt#5**  **GGT**  **Inferior Motor** | **Pt#6**  **PiD**  **Dorsolateral Prefrontal** |
| --- | --- | --- | --- |
| WM Subj./Overall | 0 | -1 | +2 |
| WM UF/Subj. | -2 | 0 | -1 |
| Upper/Lower Layers | +1 | +1 | 0 |
| BoB/GM | -2 | a | 0 |
| GM/Subj. WM | +2 | +2 | +1 |
| Atyp. Features | UB | MB++, WS | MB |

TDPA= FTLD-TDP subtype A, TDPC= FTLD-TDP subtype C, GGT= FTLD-tau with globular glial tauopathy, PSP= FTLD-tau with progressive supranuclear palsy, PiD = FTLD-Tau with Pick’s disease; UB = upper-cortical hypointense band, WS = striated white matter; a = BoB was completely occluded by mid-cortical hypointense band through entire gyrus

**Supplementary Table 5. T2*w MRI Image Details**

|  |  | **Discovery Cohort** | | | | | | | **Replication Cohort** | | |
| --- | --- | --- | --- | --- | --- | --- | --- | --- | --- | --- | --- |
|  | **Patient #** | **1** | **2** | **3** | **4** | **5** | **6** | **7** | **8** | **9** | **10** |
|  | Avgs | 5 | 5 | 8 |  | 5 | 5 | 13 | 5 | 5 | 5 |
|  | Matrix | 448 x 294 x 690 | 448 x 266 x 628 | 442 x 256 x 570 | 448 x 292 x 690 | 448 x 294 x 690 | 448 x 292 x 690 | 256 x 252 x 464 | 448 x 268 x 632 | 448 x 292 x 690 | 448 x 266 x 628 |
| SNR | Anterior Temporal |  | 56.22 | 51.18 | 61.31 | 96.08 | 59.09 |  | 54.55 | 82.89 |  |
|  | Orbitofrontal | 61.40 | 39.99 | 66.75 | 42.15 | 75.28 | 59.98 | 183.01 |  |  |  |
|  | Inferior Prefrontal | 80.91 | 48.70 | 106.38 | 55.63 | 67.59 | 52.60 | 96.88 |  |  |  |
|  | Primary Motor | 61.17 | 39.16 | 82.85 | 57.82 | 59.25 | 39.65 | 207.02 |  |  | 34.45 |
|  | Primary Sensory | 49.86 | 31.71 | 53.47 | 60.14 | 60.78 | 49.40 | 156.46 |  |  |  |
|  | Dorsolateral Prefrontal |  |  |  |  |  |  | 46.28 |  |  |  |
|  | Inferior Motor |  |  |  |  | 43.18 |  |  |  |  |  |
|  | Lateral Orbitofrontal |  |  |  | 38.25 |  |  |  |  |  |  |

Avgs: number of signal averages acquired. Matrix: imaging matrix in pixels. Regional SNR values computed by selecting an ROI containing ~100 voxels and computing mean image intensity within the cortex ($M_{tissue}$) and in the fomblin immediately proximal to the tissue ($M_{background}$). Noting that images result from root mean square combination of two channels, and assuming circular complex Gaussian noise in each channel, we can compute the noise standard deviation from the background mean intensity $M_{background}$ via the approximation $\sigma\approx M_{background}/1.88$, and thus $\mathrm{SNR}=1.88 M_{tissue}/M_{background}$.

**Supplementary Figure 1. Myleoarchitecture of cortex in healthy control hemisphere.** In four representative regions we show T2*w MRI, co-located serial sections stained with iron and LFB (myelin) stain, and high-mag zoomed images of iron and LFB (myelin) stain from the deep cortical GM these same slices. Bands of Baillarger are visible as hypointense tangential stripes in the middle layers of the cortex, while radial fibers produce a moderate hypointensity of lower-layer cortical GM relative to more hyperintense upper layers. The myeloarchitecture varies from region to region, producing specific localized laminar pattern of T2*w image contrast.

**Supplementary Figure 2. Pathological T2* signal in FTLD-Tau cases does not correspond to co-morbid amyloid plaque pathology.** Photomicrographs depict semi adjacent sections from 4R tauopathy (GGT, patient #5; Thal phase A2 CERAD C0) and progressive supranuclear palsy 4R tauopathy (PSP, patient #6; Thal phase A2 CERAD C3) stained for iron (FE) and amyloid-beta (AB). Top row of histology sections depicts 1x magnification and bottom rows depicts 32 x high magnification views from pathological areas (boxes). Pathological iron in mid to deep layers (arrows) corresponds to clusters of iron-rich microglia and not to the mild amounts of amyloid plaque pathology that was absent in these areas, suggesting patterns observed in MRI of these cases is driven by pathophysiology of 4R tauopathy and not mild plaque co-pathology.

**Supplementary Figure 3. Comparison of DAB-enhanced Perl’s iron staining and ferritin immunostaining in discovery cohort.** Photomicrographs depict iron-stained sections from regions of abnormal T2*w MRI signal in FTLD and semi-adjacent sections immunostained for ferritin. The healthy control sample had iron reactivity largely limited to oligodendrocytes and healthy white matter with only rare iron- or ferritin-positive microglia. In FTLD samples the iron-rich microglia (black arrowheads) were similarly detected in ferritin immunostaining, while iron rich processes corresponding to astrocytes (open arrowheads) were minimally detected with ferritin immunostaining, likely due to known minimal ferritin content in astrocytes compared to microglia and oligodendrocytes.

**Supplementary Figure 4. Histopathological comparison of iron-rich glia in samples from left and right hemispheres.** Photomicrographs depict iron-stained sections from standard sampled regions from each hemisphere, including the ipsilateral region with abnormal T2*w MRI signal and corresponding contralateral hemisphere for patients with available tissue which finds similar patterns and severity of iron-rich gliosis in FTLD across hemispheres.
